# Epidemiological Tracking and Population Assignment of the Non-Clonal Bacterium, *Burkholderia pseudomallei*

**DOI:** 10.1371/journal.pntd.0001381

**Published:** 2011-12-13

**Authors:** Julia Dale, Erin P. Price, Heidie Hornstra, Joseph D. Busch, Mark Mayo, Daniel Godoy, Vanaporn Wuthiekanun, Anthony Baker, Jeffrey T. Foster, David M. Wagner, Apichai Tuanyok, Jeffrey Warner, Brian G. Spratt, Sharon J. Peacock, Bart J. Currie, Paul Keim, Talima Pearson

**Affiliations:** 1 Center for Microbial Genetics and Genomics, Northern Arizona University, Flagstaff, Arizona, United States of America; 2 Menzies School of Health Research and Northern Territory Clinical School, Royal Darwin Hospital, Darwin, Northern Territory, Australia; 3 Department of Infectious Disease Epidemiology, Imperial College London, London, United Kingdom; 4 Mahidol-Oxford Tropical Medicine Research Unit, Faculty of Tropical Medicine, Mahidol University, Bangkok, Thailand; 5 Microbiology and Immunology, School of Veterinary and Biomedical Sciences, James Cook University, Townsville, Queensland, Australia; 6 Department of Microbiology and Immunology, Faculty of Tropical Medicine, Mahidol University, Bangkok, Thailand; 7 Department of Medicine, University of Cambridge, Cambridge, United Kingdom; 8 Pathogen Genomics Division, Translational Genomics Research Institute, Phoenix, Arizona, United States of America; George Washington University, United States of America

## Abstract

Rapid assignment of bacterial pathogens into predefined populations is an important first step for epidemiological tracking. For clonal species, a single allele can theoretically define a population. For non-clonal species such as *Burkholderia pseudomallei*, however, shared allelic states between distantly related isolates make it more difficult to identify population defining characteristics. Two distinct *B. pseudomallei* populations have been previously identified using multilocus sequence typing (MLST). These populations correlate with the major foci of endemicity (Australia and Southeast Asia). Here, we use multiple Bayesian approaches to evaluate the compositional robustness of these populations, and provide assignment results for MLST sequence types (STs). Our goal was to provide a reference for assigning STs to an established population without the need for further computational analyses. We also provide allele frequency results for each population to enable estimation of population assignment even when novel STs are discovered. The ability for humans and potentially contaminated goods to move rapidly across the globe complicates the task of identifying the source of an infection or outbreak. Population genetic dynamics of *B. pseudomallei* are particularly complicated relative to other bacterial pathogens, but the work here provides the ability for broad scale population assignment. As there is currently no independent empirical measure of successful population assignment, we provide comprehensive analytical details of our comparisons to enable the reader to evaluate the robustness of population designations and assignments as they pertain to individual research questions. Finer scale subdivision and verification of current population compositions will likely be possible with genotyping data that more comprehensively samples the genome. The approach used here may be valuable for other non-clonal pathogens that lack simple group-defining genetic characteristics and provides a rapid reference for epidemiologists wishing to track the origin of infection without the need to compile population data and learn population assignment algorithms.

## Author Summary

*Burkholderia pseudomallei* is a soil-dwelling bacterium that can infect a large range of hosts. In humans, *B. pseudomallei* causes melioidosis, and typical routes of entry include open wounds, inhalation, or ingestion. Clinical features are diverse, although pneumonia and abscess formation are common. High rates of recombination within the genome of this bacterium have confounded attempts to match clinical samples to geographically defined populations. Here we provide a reference that simplifies source attribution issues. We applied population assignment software to previously generated sequence data from seven *B. pseudomallei* genes to define the major geographic populations within this species. We evaluated the robustness of our results by comparison with two additional population assignment programs. We present the likelihood that each variant is assigned to a particular geographic population. This information can be used to assign novel *B. pseudomallei* isolates to a geographic population without needing to learn and run cumbersome population assignment applications. This method can also be used for other bacteria that are difficult to source-attribute due to high levels of genomic variation and recombination.

## Introduction

*Burkholderia pseudomallei*, the etiologic agent of melioidosis, is commonly isolated from soil and water in many tropical regions of the world. Endemic foci of *B. pseudomallei* predominantly include Southeast Asia (particularly Thailand) and northern Australia, although this organism is found sporadically in other equatorial regions such as South and Central America, Africa, and the Indian subcontinent [1]. Since infections are most commonly acquired from the environment, genetic differentiation is expected to occur, leading to geographic substructure within the bacterial population. Previous studies have demonstrated that *B. pseudomallei* populations from the melioidosis-endemic regions in Southeast Asia and Australia are not only geographically distinct but exhibit differences in clinical presentation and genetic features [2], [3], [4]. For example, differences in clinical manifestations include parotid abscesses, which are much more prevalent in Thailand (15%) than Australia (4%). In contrast, genitourinary infections and brainstem encephalitis are more commonly seen in Australia than Thailand (15% vs. 2% and 2% vs. <0.2%, respectively) [4], [5]. Differences in mortality rates also differ greatly between the two endemic regions, with mortality rates of approximately 50% in Thailand compared with <20% in Australia [5]. The difference in mortality rates could reflect differences in virulence but is probably more likely to be due to differences in intensive care provisions between the two regions [6]. Despite these marked differences, none are fully diagnostic for isolates from specific geographical regions.

Multilocus sequence typing (MLST) [7] is a bacterial genotyping method that involves the comparison of ∼450 bp-long nucleotide sequences from seven housekeeping genes. An MLST scheme has been developed for *B. pseudomallei* [8] and 699 sequence types (STs) from isolates and multiple species (as of November 6^th^, 2010) populate the public database (http://bpseudomallei.mlst.net/). These data have shed light on the population structure of this species. It has been previously observed that *B. pseudomallei* STs from Australia and Southeast Asia are mutually exclusive as phylogenetic analyses show geographically correlated clusters of STs, although these analyses failed to group all samples from either region together [9] [10]. Due to relatively low levels of sequence diversity and high levels of lateral gene transfer among *B. pseudomallei* isolates [8], [11], sequence data from only seven genes are insufficient for robust phylogenetic discrimination [11], [12]. Pearson *et al.* therefore used a population genetics approach to determine that *B. pseudomallei* STs form two distinct populations, conforming to the geographic regions of Southeast Asia and Australia [11]. Despite the phylogenetic limitations of MLST data, this large public database shows potential for population assignment using population genetic analyses.

We further evaluate and update the previous population assignments [11] by comparing these results with commonly used assignment algorithms. The program *Structure* [13] is a Bayesian-based clustering algorithm that has been used to infer population structure within genetically diverse bacteria such as *Helicobacter pylori* [14]. Comparison of *Structure* with other population assignment software allowed us to assess the robustness of our population assignments. The *B. pseudomallei* population assignment results that we provide, along with a probability estimation of each assignment, can be used as a practical and immediate reference for melioidosis researchers interested in identifying geographic origins of *B. pseudomallei* STs and may serve as a model for other weakly clonal species.

## Methods

### MLST dataset

The data used to define populations and evaluate the robustness of population assignments were downloaded from the *B. pseudomallei* MLST database (http://bpseudomallei.mlst.net/) on January 15^th^, 2009. The database consisted of 641 *B. pseudomallei* STs from 1802 isolates collected over 89 years from 35 countries. Approximately 44% of these isolates were collected in Southeast Asia and 53% in Australia and Papua New Guinea. The data were downloaded again on November 9^th^, 2010, in order to provide more updated population assignments and population allele frequencies for all currently known STs. These most recent data consist of 664 STs from 1829 isolates, where 44% of these isolates were collected in Southeast Asia and 53% of the isolates were collected in Australia and Papua New Guinea. More detailed information on the geographical sources of isolates representing each ST can be found in the profiles datasheet in the MLST database.

### Population analyses using *Structure*

The program *Structure* [13] (versions 2.2–2.3.1 due to software updates over the course of this study) was used to analyze allelic profile data from the original 641 *B. pseudomallei* STs. Briefly, *Structure* uses MLST datasets and a Bayesian approach to identify population structure and to assign individuals to populations without *a priori* population descriptions. A Markov Chain Monte Carlo simulation of 100,000 iterations with a burn-in period of 30,000 was run to determine the posterior probability of the number of populations (*K*). Where *K* = 2–4, *Structure* analyses were repeated eight times and the posterior probabilities from each run were averaged. For populations of *K* = 5–17, *Structure* analyses were repeated three times and the posterior probabilities averaged. Fewer repetitions were carried out for these higher *K* values as previous work suggests that more populations are not well supported [11]. The most statistically supported *K* value was selected to represent the number of populations among the STs based on the estimated log (*ln*) of the probability of the data (*ln P(D)*), and the variance exhibited by each *K* value. All simulations were carried out using both the “no admixture” [15] and “admixture” models [16] (comparison between these two models is shown in Supplemental Data Figure S1). The posterior probability of the data (*ln P(D)*) for a given value of *K* might be expected to peak at the true value of *K*, however, in our runs there was no definite peak as *ln P(D)* increased slightly with an increase in *K*. This pattern, along with an increase in the variance of *ln P(D)* is common and has been reported by Evanno and colleagues [17] who suggest that measuring the changes in likelihood is a more accurate method for estimating the true value of *K*. We therefore used Δ*K* to determine the optimal *K* value of the *B. pseudomallei* populations. The Δ*K* value corresponds with the second order rate of change of all *K* values divided by the standard deviations from each *K* [17]. Calculation of Δ*K* is shown in Supplemental Data Text S1.

### Population analyses using BAPS

We used both BAPS and *Structure* results to assess population assignments [18]. BAPS (version 4) is another free software package for Bayesian inference of genetic structure within a given dataset [19], [20], [21], [22]. Using the “clustering of linked loci” module, BAPS determines the log likelihood in 10% increments of different population divisions and subsequently calculates the most likely *K* value. Thus, unlike with *Structure*, *K* is not selected *a priori*. The likelihood of population assignment for each ST is also calculated by BAPS. For BAPS analyses, we used sequence data from the seven *B. pseudomallei* MLST loci. The codon linkage model and an upward bound of 20 populations were chosen for the “clustering of linked loci” module. As with *Structure*, eight iterations were run where *K* = 2–4 and three iterations were run where *K* = 5–17.

### Assessment of *Structure* and BAPS population assignments

As there is no empirical measure of determining the accuracy of population assignments, we further assessed *Structure* and BAPS assignments of *B. pseudomallei* using MLST data, by comparing individual ST assignments made by *Structure* and BAPS to the geographic information listed in the MLST database and to the likelihood of assignment into each population as calculated by Genetic Analysis in Excel (GenAlEx) v.6 [23]. We also used GenAlEx to measure the degree of population differentiation among populations defined by *Structure* and BAPS.

GenAlEx is a free Microsoft Excel add-in where datasets can be analyzed and manipulated without the requirement for multiple programs. We used the population assignment method in GenAlEx to determine the likelihood of inclusion in each population for each ST. Unlike *Structure* and BAPS, GenAlEx requires *a priori* population designations to define population allele frequencies and subsequently calculate the likelihood of population assignment for each ST. We compared the population assignment results from our *Structure* and BAPS results to the likelihood of population assignment calculated by GenAlEx. Also, for population defined by *Structure* and BAPS, we performed analyses of molecular variance (AMOVA) to calculate the degree and statistical significance of population differentiation.

### Characterization of *B. pseudomallei* populations

The number of populations supported by *Structure* and BAPS are two and three respectively. We therefore used the results from the *Structure* run with the highest likelihood score at *K* = 2 and the BAPs run with the highest likelihood score at *K* = 3 to infer population assignments for each ST. To show the extent of genetic differentiation among these populations, we used GenAlEx [24] to calculate Φ_PT_, using 999 permutations [23]. In assessing assignment results, we categorized STs according to the likelihood of assignment of each ST into a population by *Structure* or BAPS, allowing us to evaluate the effect of assignment confidence on discrepancies among programs. To be conservative in our assignment of STs to a population, we suggest that a ST only be considered to be from a given population if *Structure* or BAPS assigned it to that population ≥95% of the time. As BAPS measures likelihoods in 10% intervals, this threshold is effectively 100% for BAPS. STs assigned to either population <95% of the time were considered “undefined” even though studies using simulated datasets suggest that in some situations, assignment probabilities of >50% may be accurate [18].

### Construction of allele frequency charts

We wished to provide researchers interested in *B. pseudomallei* population genetics with a tool for population assignment in instances where novel STs not included in this study are encountered. To achieve this goal, the frequencies of alleles belonging to STs from each population for >95% of the runs were determined. We also enumerated alleles for STs assigned to a population between 50 and 95% of the time as this measure can be useful for indicating the reliability of an allele for population assignment.

### Definition of *B. pseudomallei* populations with single-nucleotide polymorphisms

Performing MLST on large bacterial collections is a time-consuming task; however, single nucleotide polymorphism (SNP) genotyping provides a streamlined way to characterize MLST populations even for recombining species [25], [26], [27]. We predicted that SNPs within MLST loci could be used to distinguish between the major *B. pseudomallei* ST populations. The program ‘Minimum SNPs’ [26], with incorporated Not-N algorithm [28], was used to search for a set of highly informative characters among the MLST alignments that could be used to distinguish between a predefined ‘ingroup’ and the remaining ‘outgroup’ population. The 566 *B. pseudomallei* STs determined by *Structure* to be assigned to one of the two populations in ≥95% of iterations were tested using the Not-N algorithm, where each population was alternately considered the ‘ingroup’ and all other STs the ‘outgroup’. Similarly, the 607 *B. pseudomallei* STs identified by BAPS as belonging to any of the three populations in ≥90% of iterations were tested (BAPS measures likelihood in 10% increments). In an attempt to increase the likelihood of finding a small set of population-defining SNPs, a second ‘Minimum SNPs’ analysis including only the 413 STs assigned to a population in 100% of *Structure* runs and a third analysis with the 560 STs assigned to a population in 100% of BAPS runs were carried out.

## Results and Discussion

### Population assignment of *B. pseudomallei* STs using *Structure*

**Figure 1:**
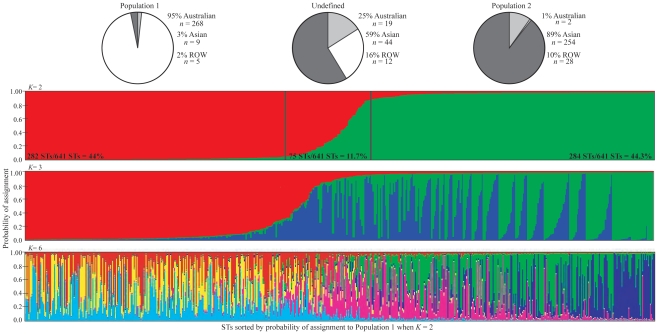
Estimated population assignments of *B. pseudomallei* genotypes based on multilocus sequence typing data and *Structure*. Each thin vertical line represents one sequence type (ST) and is divided into *K* portions (based on color) that represent the likelihood of assignment into *K* populations. STs are sorted by probability of assignment into Population 1 (predominantly Australian STs) when *K* = 2. Two black vertical lines show thresholds of 95% probability of assignment. We considered STs with assignment probabilities below these thresholds to be “undefined”. The pie charts indicate the geographical sources of STs that comprise each group. Rest-of-the-world (ROW, shown in light grey) is composed of STs that were isolated from regions other than Australia (illustrated as the white slice) or Southeast Asia (shown as the dark grey slice) according to the public MLST database (http://bpseudomallei.mlst.net/).

*Structure* was used to identify and characterize *B. pseudomallei* populations using MLST allelic profile data from 641 STs. The existence of two *B. pseudomallei* populations (*K* = 2) was first proposed by Pearson and coworkers [11] as higher values of *K* did not break apart the two main populations and subdivisions were inconsistent between runs. Here, we confirm that when using *Structure*, two populations (*K* = 2) garners the most statistical support when compared to other numbers of putative populations (*K* = 1, and 3 through 17). This support is based on three criteria that have been used in other studies to justify selected *K* values. First, higher values of *K* retained the two populations (Figure 1) [11]. Second, the selected *K* value has the lowest variance of *ln P(D)* after *K* = 1 (Figure S2) [13]. Lastly, the Δ*K* shows a peak at the selected *K* value (Figure S2) [17]. We also tested both ‘admixture’ and ‘no admixture’ analyses and obtained the same results regarding the size of *K* and similar results regarding population assignments for individual STs. However, the ‘no admixture’ method provided more consistent results than the ‘admixture’ approach, yielding lower variances. The results presented here are from the “no admixture model” (see Figure S1 for a comparison of these tests).

Using a *K* = 2 with *Structure*, the two populations were significantly distinct (Φ_PT_ = 0.123; *P* = 0.001). *Structure* assigned 88.3% of STs to either Population 1 or Population 2 with ≥95% probability of assignment, with 44% and 44.3% of STs assigned to Population 1 and 2, respectively (Figure 1). Population 1 is comprised of 95% Australian (Australia and Papua New Guinea), 3% Southeast Asian, and 2% STs from the other parts of the world. In contrast, 89% of STs in Population 2 are from Southeast Asia, 1% from Australia, and 10% from the rest of the world (Figure 1). Only 11.7% of STs were not assigned to a given population based on a 95% probability of assignment threshold. This “undefined” group is comprised of STs from Southeast Asia (59%), Australia (25%), and the rest of the world (16%).

### Population assignment of *B. pseudomallei* STs using BAPS

We also used the population-clustering program BAPS for determining the number of *B. pseudomallei* populations and for assigning STs to each population. Unlike *Structure* we used concatenated MLST sequence data rather than the allelic data used in *Structure*. In BAPS, the estimated number of populations with the most statistical support was *K* = 3 rather than *K* = 2 determined by *Structure*. This third population defined by BAPS appears to be a sub-population of the previously identified Population 2; however, other than a mostly Asian origin, we found no geographic or epidemiological correlation among these subdivided Population 2 STs. We therefore refer to these two BAPS Asian populations as Population 2a and Population 2b. Evidence of this population subdivision was also observed in *Structure* when *K* = 3 (Figure 1); however in *Structure*, both Population 1 and Population 2 were alternately subdivided depending on the run and assignments of STs to either sub-population were inconsistent. In BAPS however, Population 2 is consistently subdivided and ST assignments are consistent among runs. Therefore, it is possible that further sub-structure exists in the *B. pseudomallei* populations, but remain unresolved due to the limitation of having only seven MLST loci, which may not provide the genetic resolution to detect further subdivision.

### Comparing *Structure* and BAPS population assignments

**Figure 2:**
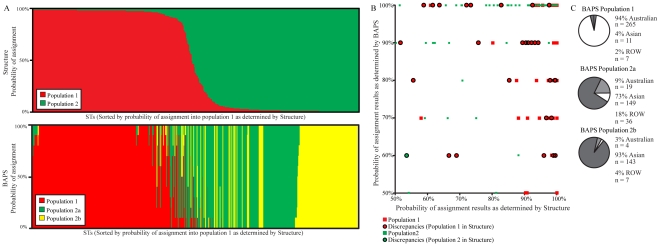
Estimated population assignments and comparisons using BAPS and *Structure* simulations. (A) Likelihood of ST assignment into two populations by *Structure* (top) and three populations by BAPS (bottom). The order of STs in both plots are the same and sorted by probability of assignment into Population 1 by *Structure*. Each thin vertical line represents one ST and is divided into two and three portions (for *Structure* and BAPS respectively) that represent the likelihood of assignment into each population. (B) A comparison of *Structure* and BAPS results. STs placed by both programs into Population 1 are shown in red and Population 2 (Populations 2a and 2b given by BAPS) are represented in green. The discrepant assignments by the two programs are shown as circles where a red interior denotes assignment into Population 1 by *Structure* and a green interior denotes assignment into Population 2 by *Structure*. (C) A breakdown of BAPS Populations 1, 2a, and 2b according to BAPS results and source data on the MLST database. The white region denotes Australian STs, the light grey region represents the ROW STs, and the dark grey color represents the Southeast Asian STs.

We compared the population assignments made by the run with the highest likelihood from *Structure* (*K* = 2) and BAPS (*K* = 3) (Figure 2). As BAPS Populations 2a and 2b are essentially subpopulations of *Structure* Population 2, we searched for discrepant STs assigned to Population 1 with >50% likelihood by one program and Population 2 with >50% likelihood by the other. Of the 29 discrepancies (Figure 2B), 16 were assigned by either program with a confidence level ≥95% (one ST was assigned by both programs with a confidence level ≥95%). As a further measure of assignment accuracy, we compared these 16 discrepant STs to the geographical data listed in the MLST database. Eight of the nine discrepancies assigned to a population ≥95% using *Structure* matched the geographical data listed in the MLST database. For the discrepancies assigned to a population ≥95% with BAPS, 3/8 originated from the geographical region of the population assigned by BAPS. Even though the listed geographic source of a ST is not a perfect indicator of population, it is possible that both programs make assignment errors even when confidence values are >95%, however such errors are probably rare. The geographic sources of STs that comprise each BAPS population are shown in Figure 2C.

### Comparison of *Structure* and BAPS population assignments with GenAlEx

**Figure 3:**
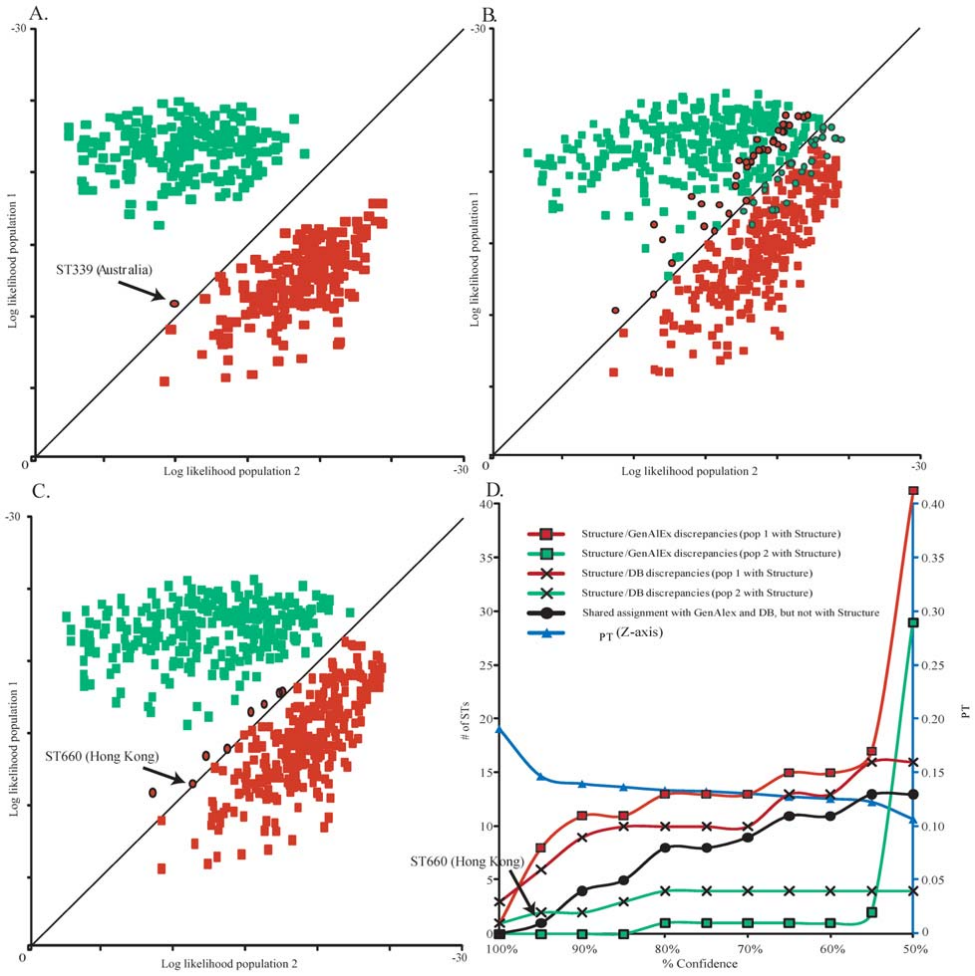
Population assignments of STs using GenAlEx and assignment discrepancies with *Structure*. Charts (A–C) represent the log likelihood of assignment of each ST by GenAlEx. *A priori* population designations were made with *Structure* and those STs assigned to a population in 100% of iterations (A), ≥95% of iterations (B), and ≥50% of iterations (C). STs with *a priori* designation as Population 1 are shown in red while those designated as from Population 2 are shown in green. STs with a log likelihood of assignment as calculated by GenAlEx that was in disagreement with *Structure* assignments are outlined in black. See text for a discussion on ST339 and ST660 indicated in A and C. (D) The relationship between % confidence and discrepancies between *Structure* and GenAlEx, between *Structure* results and published origin in the MLST database, and with the estimate of the population genetic differentiation (Φ_PT_).

**Figure 4:**
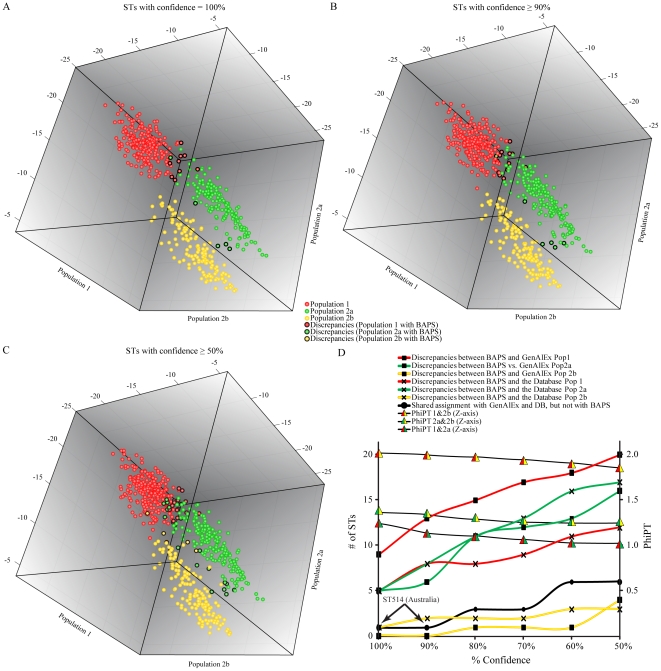
Population assignments of STs using GenAlEx and assignment discrepancies with BAPS. STs from the three (*K* = 3) populations identified by BAPs were assigned to three populations in GenAlEx. Charts (A–C) represent the log likelihood of assignment of each ST by GenAlEx. *A priori* population designations were made with BAPS and those STs assigned to a population in 100% of iterations (A), ≥95% of iterations (B), and ≥50% of iterations (C). STs with *a priori* designation as Population 1 are shown in red, Population 2a STs are shown in green, and Population 2b are shown in yellow. STs with a log likelihood of assignment as calculated by GenAlEx that are in disagreement with BAPS assignments are outlined in black. Some discrepancies may not be visible due to the three-dimensional structure of the figure. (D) The relationship between % confidence and discrepancies between BAPS and GenAlEx, discrepancies between BAPS results and published origin in the MLST database, and with the estimate of the genetic differentiation between populations (Φ_PT_).

To further evaluate *Structure* and BAPS assignments, we used GenAlEx to calculate the likelihood of assignment of each ST in each population. When STs with high probabilities of assignment using either *Structure* or BAPS were analyzed with GenAlEx, a more distinct differentiation of populations could be seen (Figures 3 & 4) and the likelihood calculations from GenAlEx placed only a few STs in a different population than *Structure* or BAPS. As expected, differentiation among populations eroded (reflected in a decline of Φ_PT_ values) and the number of discrepancies between either Structure or BAPS and GenAlEx increased as STs with lower assignment probabilities from *Structure* or BAPS were analyzed with GenAlEx (Figures 3 & 4).

When only STs with 100% probability of assignment in *Structure* were analyzed with GenAlEx, there was only one discrepancy (ST339). We confirmed that ST 399 is an environmental isolate from the Darwin region of the Northern Territory, Australia. *Structure* assigned this ST to Population 1, as expected, but was given a higher likelihood of belonging to Population 2 by GenAlEx (Figure 3A & 3D). When STs with ≥95% probabilities of assignment with *Structure* were analyzed with GenAlEx, there were eight discrepancies. These discrepant STs clustered with STs from Population 1, despite log likelihood values from GenAlEx that suggested they belonged in Population 2, albeit with little difference in log likelihood values (Figure 3B). The geographic sources of these eight discrepancies suggest that only one ST may have been erroneously assigned by *Structure*; specifically, ST660 is from rain water in Hong Kong and would be expected to be in Population 2, whereas the other seven were from Northern Australia which is consistent with their position within Population 1. As STs with decreasing probabilities of assignment with *Structure* were analyzed with GenAlEx, the number of discrepancies increased slightly, except for a large increase when all STs were analyzed (Figure 3).

Interestingly, more discrepancies occurred with Population 1 than Population 2. It has been previously observed that the Southeast Asian *B. pseudomallei* population (i.e. Population 2) has high levels of recombination but low allelic diversity, due to a monophyletic introduction of *B. pseudomallei* into Southeast Asia. In contrast, the Australian population appears to be paraphyletic with greater allelic diversity in spite of lower recombination between STs [11]. Therefore, the greater diversity of Australian alleles may make Bayesian assignment of STs into Population 1 more complex than Population 2. Our cut-off value of ≥95% is likely to result in very few erroneous assignments using *Structure*. Indeed, ST660 is the only potentially inaccurate assignment that we identified at this cut-off value.

When GenAlEx was compared against the BAPS *K* = 3 dataset, there were 14 discrepancies when only STs with 100% probability of assignment were analyzed with GenAlEx (Figure 4A & 4D). For only one of these discrepancies (ST514), the assignment by BAPS into Population 2a is not consistent with the geographic origin listed in the MLST database (Australia), representing a potentially erroneous assignment by BAPS. Four STs were assigned to Population 2a by one program and Population 2b by the other. As geographic correlates for these two populations are unknown, it is impossible to determine which assignment is more likely. For the remaining nine discrepancies between BAPS and GenAlEx, the geographic origin listed in the MLST database is consistent with the BAPs population assignment. When STs at the ≥90% assignment probability with BAPS were analyzed with GenAlEx, there were 19 discrepancies. Two of these discrepancies (ST 514 and ST 660) are likely erroneous assignments by BAPS into Populations 2a and 1 respectively as their geographic origins as listed in the MLST database are Australia and Hong Kong, respectively. The number of discrepancies continues to rise as more STs are analyzed and the threshold for inclusion drops to ≥50% assignment probability with BAPS. At all levels of assignment probability by BAPS, most discrepancies involved assignments by BAPS into Population 1 while few discrepancies occurred with STs assigned by BAPS into Population 2b. This is similar to the pattern of discrepancies found with *Structure* assignments. This observation suggests that assignments into Population 1 are the most challenging while assignments into Population 2b are least difficult and probably more robust. In comparison to the *Structure*-GenAlEx comparisons, there were more overall discrepancies for GenAlEx and BAPS; however, this was expected as BAPS is splitting STs into three populations rather than just two.

In addition to evolutionary dynamics and computer algorithms, discrepant population assignment of certain STs can occasionally be attributed to database errors. Indeed, it has been shown that the listed origins for some *B. pseudomallei* STs are not always accurate due to curation difficulties or by not being able to account for patient travel histories. For example, several isolates recovered in the USA were likely from infections acquired during travel in Southeast Asia [10]. Using our population assignment data, we have identified and corrected some database errors, however, it is possible that other errors remain. There are discrepancies between *Structure* and BAPS assignments and the listed origin of a ST in the MLST database. We therefore paid particular attention to those STs where both GenAlEx and the MLST database suggested a different population assignment than *Structure* (Figure 3D) or BAPS (Figure 4D). At the 95% likelihood level for *Structure*, only one such discrepancy (ST660) exists. Although erroneous attribution must always be considered, it is possible that this ST is derived from a recent, but ecologically established introduction into Hong Kong. Another possibility is that this ST was erroneously assigned by *Structure* to Population 1. However, BAPS similarly assigned ST660 to Population 1 albeit with 82% likelihood. At or above the 95% likelihood level, we could therefore find only one potential example of an inappropriate assignment by *Structure*.

At ≥90% likelihood level for BAPS, we found one potential discrepancy when compared to *Structure*, the MLST database and GenAlEx. Sequence type 514 was assigned by *Structure* at 100% confidence in Population 1. However, BAPS assigned ST514 with 100%confidence into Population 2a. The MLST database lists ST514 as being collected from a human source in Australia. Unfortunately, this information does not confirm the origin since travel between Thailand and Australia is prevalent. Whole genome sequencing of this ST will help resolve uncertainties regarding Australian and Southeast Asian population assignments as phylogenetic analyses can be expected to reflect population subdivisions as they have for the Australian and Southeast Asian populations [11].

Of the discrepancies between *Structure* and BAPS versus GenAlEx, the *Structure* results were most closely aligned with the geographical origin of STs as listed in the MLST database. However, both BAPS and GenAlEx were able to identify instances where *Structure* population assignments were inconsistent with the epidemiological data, indicating that no single program was 100% effective in *B. pseudomallei* ST population assignment. Therefore, we suggest, where possible, that *Structure* and BAPS are used in concert with large epidemiological datasets for highly recombinant organisms to make the most robust population assignments. The addition of more loci and more thoroughly sampling isolates not assigned to either population with high confidence will likely lead to a better understanding of the intricacies of *B. pseudomallei* population structure.

### The search for population-defining SNPs

Given the genetic delineation of up to three populations using population assignment software, we hypothesized that a combination of SNPs might be identified that readily differentiate between these *B. pseudomallei* populations. We used the program ‘Minimum SNPs’, with incorporated Not-N algorithm [28], to find population-specific SNPs from both *Structure* and BAPS defined populations. Using STs with ≥95% population assignment from *Structure*, we identified a set of 25 SNPs that were needed to discriminate STs from Population 2 from all other STs, albeit with a confidence of only 92.5%. In other words, even with a set of 25 SNPs, only 92.5% of the Population 2 STs could be distinguished from the Population 1 STs. No addition SNPs could be added by the algorithm to increase the percentage of Population 2 STs that could be distinguished from Population 1 STs. In order to increase the likelihood of identifying a smaller number of SNPs for population differentiation, we narrowed down our population definition by including only STs assigned to each population in 100% of *Structure* runs. Our results showed that a set of 16 SNPs were needed to separate the Population 2 STs from the Population 1 STs at a confidence level of 97.6%. As inaccurately assigned STs can hamper the ability of ‘Minimum SNPs’ to find population specific SNPs, we also used the BAPS population designations at both the ≥90% and 100% thresholds for population assignment. For STs assigned to each population in 90% of BAPS runs, the Not-N algorithm identified a set of 26 SNPs that discriminated Populations 2a and 2b apart from Population 1 with a confidence of 81.1%. For STs assigned to a given population in 100% of BAPS runs, a set of 26 SNPs discriminated Populations 2a and 2b apart from Population 1 with 84.3% confidence. A set of 21 SNPs discriminated Population 2a apart from Populations 1 and 2b with 95.5% confidence while a set of 13 SNPs discriminated population 2b from the others with 99.2% confidence. Finally, by analyzing only the Population 2 STs identified at the 100% threshold with BAPS, we found a single SNP (at position 192 in the narK locus) that distinguishes all STs in Population 2b (C nucleotide) from all STs in Population 2a (G or T nucleotide). These results suggest that complete population identification of all members of all populations by a combination of SNPs from MLST data is not possible.

### Development of reference tools for population assignment

A more recent version of the MLST database was downloaded and used to repeat our *Structure* and BAPS analyses. Once the analyses on the updated database were complete (November 6^th^, 2010) these data were compared to the database originally downloaded for this study (January 15^th^, 2009). This comparison verified the consistency of *Structure* and BAPS results between the temporal datasets. Of note, however, is the identification by BAPS of a fourth population consisting of three STs, two of which were included in the original database and were formerly placed in Population 1. The third ST in this new population (ST698) is novel and is a human isolate from the USA. Because this population appears to be part of the Australian population, we refer to it as Population 1b and the other Australian population as Population 1a.

Population assignments and likelihood values for each ST based on the updated MLST database are shown in Table 1. This table provides a resource that can be used by researchers interested in determining the geographic source population of *B. pseudomallei* STs. Comparisons with other population assignment methods and with geographic source information listed on the MLST database suggest that the risk of assignment by *Structure* and BAPS into the incorrect population is low when a high percentage of iterations result in the same assignment. In addition, there appear to be fewer potential errors with STs assigned to Population 2 by *Structure* and 2a or 2b by BAPS. We therefore suggest that a cut off value of ≥95% (≥90% for BAPS) assignment probability can serve as a conservative threshold above which assignment errors are not likely and which include a large proportion (∼90%) of the entire ST populations. The threshold used by different investigators does not need to be universal, and our recommendation of ≥95% is solely intended as a conservative guide. Indeed, for STs assigned to Population 2 (or 2a/2b), which is a monophyletic population, it is likely that a lower threshold of even ≥60% assignment probability is not likely to result in erroneous assignments.

**Table 1:** Population assignment for each ST and likelihood of assignment.

	*Structure*		BAPS					*Structure*		BAPS			
ST	Pop1	Pop2	Pop1a	Pop1b	Pop2a	Pop2b	ST	Pop1	Pop2	Pop1a	Pop1b	Pop2a	Pop2b
1	95%	5%	7%	0%	93%	0%	62	0%	100%	0%	0%	93%	6%
2	0%	100%	17%	0%	83%	0%	63	0%	100%	0%	0%	94%	6%
3	0%	100%	0%	0%	100%	0%	64	0%	100%	0%	0%	100%	0%
4	1%	100%	0%	0%	92%	8%	65	0%	100%	0%	0%	100%	0%
5	0%	100%	0%	0%	99%	1%	66	0%	100%	19%	0%	0%	81%
6	87%	14%	0%	0%	100%	0%	67	0%	100%	8%	0%	0%	92%
7	0%	100%	0%	0%	100%	0%	68	0%	100%	0%	0%	0%	100%
8	0%	100%	0%	0%	99%	1%	69	0%	100%	0%	0%	0%	100%
9	0%	100%	0%	0%	100%	0%	70	0%	100%	0%	0%	0%	100%
10	1%	99%	0%	0%	100%	0%	71	1%	100%	11%	0%	0%	89%
11	1%	99%	0%	0%	100%	0%	72	0%	100%	0%	0%	94%	6%
12	0%	100%	0%	0%	100%	0%	78	0%	100%	0%	0%	100%	0%
14	5%	95%	0%	0%	82%	18%	82	13%	87%	0%	0%	100%	0%
15	4%	96%	0%	0%	100%	0%	83	0%	100%	0%	0%	66%	34%
16	0%	100%	0%	0%	69%	31%	84	0%	100%	0%	0%	0%	100%
17	3%	97%	0%	0%	100%	0%	85	55%	45%	0%	0%	100%	0%
18	26%	74%	0%	0%	100%	0%	86	0%	100%	0%	0%	88%	12%
19	2%	98%	0%	0%	100%	0%	87	0%	100%	0%	0%	0%	100%
20	100%	0%	90%	0%	0%	10%	88	3%	97%	0%	0%	83%	17%
21	0%	100%	5%	0%	0%	95%	89	100%	0%	31%	0%	68%	0%
22	99%	1%	40%	0%	59%	1%	90	0%	100%	4%	0%	12%	84%
23	8%	92%	0%	0%	100%	0%	91	54%	47%	14%	0%	86%	0%
24	100%	0%	100%	0%	0%	0%	92	0%	100%	0%	0%	100%	0%
25	95%	5%	24%	0%	76%	0%	93	0%	100%	0%	0%	0%	100%
26	1%	99%	0%	0%	100%	0%	94	100%	0%	100%	0%	0%	0%
27	1%	99%	30%	0%	70%	0%	95	0%	100%	0%	0%	100%	0%
28	0%	100%	0%	0%	98%	2%	96	100%	0%	95%	0%	4%	0%
29	0%	100%	0%	0%	0%	100%	97	0%	100%	0%	0%	99%	1%
30	0%	100%	1%	0%	0%	99%	98	0%	100%	1%	0%	91%	7%
31	0%	100%	0%	0%	0%	100%	99	0%	100%	0%	0%	0%	100%
32	0%	100%	0%	0%	0%	100%	102	0%	100%	0%	0%	0%	100%
33	5%	95%	0%	0%	99%	1%	103	92%	8%	94%	0%	5%	0%
34	0%	100%	0%	0%	2%	98%	104	100%	0%	96%	0%	4%	0%
35	100%	0%	100%	0%	0%	0%	105	94%	6%	70%	0%	0%	30%
36	100%	0%	100%	0%	0%	0%	106	100%	0%	98%	0%	0%	1%
37	100%	0%	99%	0%	0%	0%	107	100%	0%	100%	0%	0%	0%
38	0%	100%	0%	0%	100%	0%	108	94%	6%	53%	0%	0%	47%
39	99%	1%	66%	0%	29%	5%	109	100%	0%	100%	0%	0%	0%
41	1%	99%	0%	0%	100%	0%	111	100%	0%	90%	0%	10%	0%
42	31%	69%	22%	0%	78%	0%	112	99%	1%	37%	0%	7%	56%
43	24%	76%	0%	0%	100%	0%	113	100%	0%	100%	0%	0%	0%
45	91%	9%	10%	0%	90%	0%	114	100%	0%	98%	0%	0%	2%
46	0%	100%	0%	0%	100%	0%	115	100%	0%	62%	0%	0%	38%
47	0%	100%	0%	0%	100%	0%	116	100%	0%	100%	0%	0%	0%
48	0%	100%	0%	0%	0%	100%	117	100%	0%	100%	0%	0%	0%
49	0%	100%	0%	0%	0%	100%	118	100%	0%	79%	0%	21%	0%
50	0%	100%	0%	0%	0%	100%	120	100%	0%	74%	0%	26%	0%
51	0%	100%	0%	0%	0%	100%	121	100%	0%	100%	0%	0%	0%
52	0%	100%	0%	0%	94%	6%	122	100%	0%	100%	0%	0%	0%
53	0%	100%	0%	0%	75%	25%	123	100%	0%	100%	0%	0%	0%
54	0%	100%	0%	0%	90%	10%	125	100%	0%	88%	0%	11%	0%
55	0%	100%	0%	0%	0%	100%	126	100%	0%	90%	0%	6%	0%
56	0%	100%	0%	0%	0%	100%	127	100%	0%	98%	0%	2%	0%
57	0%	100%	0%	0%	97%	3%	128	100%	0%	100%	0%	0%	0%
58	0%	100%	0%	0%	1%	99%	129	100%	0%	100%	0%	0%	0%
59	0%	100%	16%	0%	84%	0%	130	100%	0%	58%	0%	4%	37%
60	0%	100%	0%	0%	100%	0%	131	100%	0%	93%	0%	0%	7%

	*Structure*		BAPS					*Structure*		BAPS			
ST	Pop1	Pop2	Pop1b	Pop1a	Pop2a	Pop2b	ST	Pop1	Pop2	Pop1a	Pop1b	Pop2a	Pop2b
132	100%	0%	100%	0%	0%	0%	190	0%	100%	0%	0%	75%	25%
134	100%	0%	100%	0%	0%	0%	191	0%	100%	16%	0%	58%	26%
135	100%	0%	100%	0%	0%	0%	192	0%	100%	0%	0%	0%	100%
136	100%	0%	100%	0%	0%	0%	193	0%	100%	0%	0%	100%	0%
137	98%	2%	90%	0%	0%	10%	195	0%	100%	0%	0%	9%	91%
138	100%	0%	100%	0%	0%	0%	196	0%	100%	0%	0%	12%	88%
139	100%	0%	100%	0%	0%	0%	197	1%	99%	2%	0%	40%	58%
140	100%	0%	99%	0%	1%	0%	198	0%	100%	0%	0%	100%	0%
141	100%	0%	84%	0%	1%	15%	199	3%	97%	0%	0%	100%	0%
142	100%	0%	100%	0%	0%	0%	200	0%	100%	0%	0%	70%	30%
143	100%	0%	100%	0%	0%	0%	201	0%	100%	0%	0%	0%	100%
144	100%	0%	100%	0%	0%	0%	202	94%	6%	99%	0%	1%	0%
145	100%	0%	100%	0%	0%	0%	203	0%	100%	5%	0%	0%	95%
146	100%	0%	100%	0%	0%	0%	204	0%	100%	0%	0%	100%	0%
147	100%	0%	100%	0%	0%	0%	205	0%	100%	0%	0%	0%	100%
148	100%	0%	100%	0%	0%	0%	206	1%	100%	0%	0%	100%	0%
149	100%	0%	100%	0%	0%	0%	207	0%	100%	0%	0%	0%	100%
150	100%	0%	100%	0%	0%	0%	208	0%	100%	4%	0%	2%	94%
151	100%	0%	99%	0%	1%	0%	209	7%	93%	7%	0%	93%	0%
152	0%	100%	0%	0%	0%	100%	210	0%	100%	0%	0%	0%	100%
153	1%	99%	0%	0%	100%	0%	211	0%	100%	0%	0%	0%	100%
154	0%	100%	0%	0%	100%	0%	212	0%	100%	10%	0%	0%	90%
155	0%	100%	0%	0%	100%	0%	213	0%	100%	1%	0%	0%	99%
156	0%	100%	0%	0%	0%	100%	214	0%	100%	0%	0%	100%	0%
157	0%	100%	0%	0%	0%	100%	215	0%	100%	0%	0%	94%	6%
158	0%	100%	0%	0%	64%	36%	216	0%	100%	1%	0%	0%	99%
159	0%	100%	0%	0%	98%	2%	217	0%	100%	0%	0%	87%	13%
160	0%	100%	0%	0%	100%	0%	218	0%	100%	0%	0%	98%	0%
161	0%	100%	0%	0%	100%	0%	219	0%	100%	0%	0%	88%	12%
162	0%	100%	0%	0%	0%	100%	220	0%	100%	0%	0%	63%	37%
163	0%	100%	0%	0%	0%	100%	221	0%	100%	0%	0%	33%	67%
164	0%	100%	0%	0%	0%	100%	222	0%	100%	0%	0%	61%	39%
165	31%	69%	24%	0%	34%	42%	223	17%	83%	0%	0%	100%	0%
166	0%	100%	4%	0%	1%	95%	224	0%	100%	0%	0%	28%	72%
167	0%	100%	0%	0%	100%	0%	225	67%	33%	0%	0%	100%	0%
168	0%	100%	0%	0%	0%	100%	226	4%	96%	1%	0%	84%	15%
169	0%	100%	0%	0%	0%	100%	227	0%	100%	0%	0%	0%	100%
170	0%	100%	0%	0%	100%	0%	228	0%	100%	0%	0%	0%	100%
171	0%	100%	0%	0%	100%	0%	229	0%	100%	0%	0%	0%	100%
172	0%	100%	0%	0%	0%	100%	230	0%	100%	0%	0%	74%	26%
173	0%	100%	0%	0%	0%	100%	231	0%	100%	0%	0%	0%	100%
174	11%	90%	0%	0%	100%	0%	232	0%	100%	0%	0%	15%	85%
175	12%	88%	33%	0%	67%	0%	233	1%	99%	0%	0%	100%	0%
176	0%	100%	0%	0%	100%	0%	235	62%	38%	76%	0%	24%	0%
177	0%	100%	0%	0%	100%	0%	236	100%	0%	62%	0%	38%	0%
178	45%	55%	43%	0%	0%	57%	237	100%	0%	75%	0%	25%	0%
179	78%	22%	78%	0%	0%	22%	238	100%	0%	100%	0%	0%	0%
180	21%	79%	40%	0%	60%	0%	239	100%	0%	100%	0%	0%	0%
181	0%	100%	0%	0%	85%	15%	240	100%	0%	100%	0%	0%	0%
182	0%	100%	0%	0%	0%	100%	241	100%	0%	100%	0%	0%	0%
183	2%	98%	26%	0%	0%	74%	242	99%	1%	100%	0%	0%	0%
184	0%	100%	0%	0%	2%	98%	243	100%	0%	100%	0%	0%	0%
185	0%	100%	0%	0%	0%	100%	244	100%	0%	100%	0%	0%	0%
186	0%	100%	0%	0%	100%	0%	245	100%	0%	78%	0%	22%	0%
187	0%	100%	0%	0%	0%	100%	246	100%	0%	100%	0%	0%	0%
188	12%	88%	0%	0%	100%	0%	247	100%	0%	100%	0%	0%	0%
189	0%	100%	11%	0%	2%	87%	248	100%	0%	93%	0%	7%	0%

	*Structure*		BAPS					*Structure*		BAPS			
ST	Pop1	Pop2	Pop1b	Pop1a	Pop2a	Pop2b	ST	Pop1	Pop2	Pop1a	Pop1b	Pop2a	Pop2b
249	100%	0%	100%	0%	0%	0%	307	6%	94%	0%	0%	100%	0%
250	0%	100%	0%	0%	99%	1%	308	0%	100%	0%	0%	94%	6%
251	72%	28%	0%	0%	100%	0%	309	52%	48%	0%	0%	100%	0%
252	96%	4%	32%	0%	68%	0%	310	0%	100%	0%	0%	93%	7%
253	100%	0%	100%	0%	0%	0%	311	0%	100%	6%	0%	6%	87%
254	100%	0%	98%	0%	2%	0%	312	0%	100%	0%	0%	100%	0%
255	100%	0%	91%	0%	9%	0%	313	2%	98%	0%	0%	100%	0%
256	100%	0%	98%	0%	2%	0%	314	17%	83%	0%	0%	100%	0%
257	100%	0%	100%	0%	0%	0%	315	3%	97%	0%	0%	100%	0%
258	100%	0%	100%	0%	0%	0%	316	15%	85%	0%	0%	100%	0%
259	100%	0%	100%	0%	0%	0%	317	100%	0%	55%	0%	45%	0%
260	100%	0%	99%	0%	0%	0%	319	1%	99%	0%	0%	92%	7%
261	99%	1%	92%	0%	7%	0%	320	100%	0%	100%	0%	0%	0%
262	100%	0%	98%	0%	0%	1%	321	99%	1%	99%	0%	1%	0%
263	100%	0%	100%	0%	0%	0%	322	100%	0%	99%	0%	1%	0%
264	100%	0%	100%	0%	0%	0%	323	99%	1%	35%	0%	65%	0%
265	100%	0%	75%	0%	18%	6%	324	100%	0%	35%	0%	63%	1%
266	100%	0%	100%	0%	0%	0%	325	100%	0%	99%	0%	1%	0%
267	100%	0%	98%	0%	1%	0%	326	100%	0%	100%	0%	0%	0%
268	100%	0%	100%	0%	0%	0%	327	100%	0%	99%	0%	1%	0%
269	100%	0%	72%	0%	0%	28%	328	100%	0%	100%	0%	0%	0%
270	100%	0%	99%	0%	0%	0%	329	100%	0%	100%	0%	0%	0%
271	0%	100%	0%	0%	0%	100%	330	100%	0%	92%	0%	1%	6%
272	36%	64%	0%	0%	100%	0%	331	100%	0%	96%	0%	3%	0%
273	0%	100%	0%	0%	57%	43%	332	100%	0%	100%	0%	0%	0%
274	100%	0%	93%	0%	6%	1%	333	100%	0%	100%	0%	0%	0%
275	100%	0%	100%	0%	0%	0%	334	100%	0%	100%	0%	0%	0%
276	100%	0%	85%	0%	15%	0%	335	94%	7%	100%	0%	0%	0%
277	100%	0%	99%	0%	0%	0%	336	98%	2%	66%	0%	33%	1%
278	100%	0%	99%	0%	0%	0%	337	100%	0%	100%	0%	0%	0%
279	100%	0%	100%	0%	0%	0%	339	100%	0%	74%	0%	5%	21%
280	91%	9%	44%	0%	11%	45%	340	100%	0%	98%	0%	0%	1%
281	100%	0%	92%	0%	8%	0%	341	100%	0%	100%	0%	0%	0%
282	100%	0%	100%	0%	0%	0%	342	0%	100%	0%	0%	100%	0%
283	100%	0%	100%	0%	0%	0%	343	80%	20%	66%	0%	34%	0%
284	100%	0%	99%	0%	0%	0%	344	100%	0%	94%	0%	6%	0%
285	100%	0%	95%	0%	3%	0%	346	3%	97%	12%	0%	0%	88%
286	100%	0%	100%	0%	0%	0%	347	6%	94%	20%	0%	80%	0%
287	100%	0%	100%	0%	0%	0%	348	34%	66%	19%	0%	81%	0%
288	1%	99%	0%	0%	100%	0%	349	14%	86%	0%	0%	100%	0%
289	0%	100%	0%	0%	0%	100%	350	0%	100%	0%	0%	100%	0%
290	0%	100%	0%	0%	0%	100%	351	0%	100%	0%	0%	0%	100%
291	100%	0%	100%	0%	0%	0%	353	100%	0%	100%	0%	0%	0%
292	100%	0%	100%	0%	0%	0%	354	0%	100%	1%	0%	0%	99%
294	100%	0%	100%	0%	0%	0%	364	0%	100%	0%	0%	0%	100%
295	100%	0%	100%	0%	0%	0%	365	0%	100%	0%	0%	100%	0%
296	100%	0%	100%	0%	0%	0%	366	0%	100%	0%	0%	0%	100%
297	58%	43%	0%	0%	52%	48%	367	0%	100%	0%	0%	100%	0%
298	2%	98%	0%	0%	100%	0%	368	0%	100%	0%	0%	0%	100%
299	0%	100%	0%	0%	100%	0%	369	0%	100%	0%	0%	0%	100%
300	0%	100%	0%	0%	0%	100%	370	66%	34%	26%	0%	30%	44%
301	57%	43%	67%	0%	33%	0%	371	0%	100%	26%	0%	3%	71%
302	1%	99%	31%	0%	0%	69%	372	22%	78%	0%	0%	33%	67%
303	0%	100%	0%	0%	100%	0%	373	0%	100%	0%	0%	0%	100%
304	0%	100%	0%	0%	1%	99%	374	0%	100%	13%	0%	23%	64%
305	0%	100%	0%	0%	0%	100%	375	0%	100%	0%	0%	0%	100%
306	30%	70%	0%	0%	100%	0%	376	0%	100%	0%	0%	0%	100%

	*Structure*		BAPS					*Structure*		BAPS			
ST	Pop1	Pop2	Pop1b	Pop1a	Pop2a	Pop2b	ST	Pop1	Pop2	Pop1a	Pop1b	Pop2a	Pop2b
377	0%	100%	0%	0%	0%	100%	434	100%	0%	100%	0%	0%	0%
378	33%	67%	3%	0%	97%	0%	435	100%	0%	100%	0%	0%	0%
379	0%	100%	0%	0%	0%	100%	436	0%	100%	0%	0%	99%	1%
380	0%	100%	0%	0%	0%	100%	437	100%	0%	98%	0%	0%	0%
381	0%	100%	0%	0%	100%	0%	438	0%	100%	0%	0%	90%	10%
382	2%	98%	19%	0%	81%	0%	439	100%	0%	100%	0%	0%	0%
383	19%	82%	36%	0%	64%	0%	440	100%	0%	100%	0%	0%	0%
384	0%	100%	0%	0%	1%	99%	441	0%	100%	0%	0%	100%	0%
385	0%	100%	0%	0%	0%	100%	442	99%	1%	54%	0%	46%	0%
386	0%	100%	0%	0%	99%	1%	443	100%	0%	98%	0%	0%	1%
387	0%	100%	0%	0%	100%	0%	444	100%	0%	100%	0%	0%	0%
388	0%	100%	0%	0%	32%	68%	445	100%	0%	100%	0%	0%	0%
389	0%	100%	0%	0%	0%	100%	446	100%	0%	60%	0%	40%	0%
390	91%	9%	29%	0%	25%	46%	447	100%	0%	53%	0%	46%	0%
391	98%	2%	40%	0%	60%	0%	448	100%	0%	100%	0%	0%	0%
392	0%	100%	14%	0%	0%	86%	449	100%	0%	96%	0%	1%	2%
393	0%	100%	0%	0%	0%	100%	450	100%	0%	99%	0%	0%	0%
394	0%	100%	0%	0%	0%	100%	451	0%	100%	0%	0%	0%	100%
395	0%	100%	0%	0%	0%	100%	452	100%	0%	68%	0%	32%	0%
396	0%	100%	0%	0%	99%	1%	453	100%	0%	96%	0%	4%	0%
397	0%	100%	0%	0%	3%	97%	454	100%	0%	100%	0%	0%	0%
398	53%	47%	13%	0%	36%	51%	455	100%	0%	98%	0%	0%	1%
399	0%	100%	0%	0%	22%	78%	456	100%	0%	100%	0%	0%	0%
400	100%	1%	52%	0%	48%	0%	457	100%	0%	75%	0%	0%	25%
401	0%	100%	0%	0%	0%	100%	458	100%	0%	100%	0%	0%	0%
402	0%	100%	0%	0%	70%	30%	459	100%	0%	100%	0%	0%	0%
403	2%	98%	0%	0%	100%	0%	460	100%	0%	100%	0%	0%	0%
404	0%	100%	0%	0%	0%	100%	461	100%	0%	70%	0%	3%	26%
405	46%	54%	62%	0%	38%	0%	462	100%	0%	54%	0%	0%	0%
406	31%	69%	20%	0%	80%	0%	463	100%	0%	98%	0%	0%	0%
407	0%	100%	1%	0%	94%	5%	464	100%	0%	100%	0%	0%	0%
408	0%	100%	0%	0%	0%	99%	465	97%	3%	37%	0%	63%	0%
409	0%	100%	0%	0%	0%	100%	466	100%	0%	99%	0%	1%	0%
410	0%	100%	0%	0%	0%	100%	467	100%	0%	99%	0%	1%	0%
411	0%	100%	0%	0%	100%	0%	468	91%	9%	98%	0%	2%	0%
412	5%	95%	0%	0%	100%	0%	469	100%	0%	100%	0%	0%	0%
413	0%	100%	0%	0%	100%	0%	470	100%	0%	100%	0%	0%	0%
414	0%	100%	0%	0%	0%	100%	471	97%	3%	100%	0%	0%	0%
415	0%	100%	0%	0%	0%	100%	472	4%	96%	33%	0%	1%	66%
416	15%	85%	32%	0%	68%	0%	473	100%	0%	98%	0%	2%	0%
417	1%	99%	0%	0%	100%	0%	476	100%	0%	100%	0%	0%	0%
418	0%	100%	0%	0%	100%	0%	477	100%	0%	98%	0%	2%	0%
419	0%	100%	0%	0%	96%	4%	478	100%	0%	100%	0%	0%	0%
420	6%	94%	0%	0%	53%	47%	479	100%	0%	97%	0%	1%	1%
421	63%	37%	28%	0%	72%	0%	480	100%	0%	100%	0%	0%	0%
422	0%	100%	0%	0%	0%	100%	481	99%	1%	50%	0%	48%	2%
423	0%	100%	0%	0%	0%	100%	482	94%	6%	41%	0%	59%	0%
424	22%	78%	42%	0%	58%	0%	483	100%	0%	94%	0%	0%	6%
425	100%	0%	100%	0%	0%	0%	484	0%	100%	0%	0%	100%	0%
426	20%	80%	0%	0%	100%	0%	485	0%	100%	0%	0%	0%	100%
427	100%	0%	100%	0%	0%	0%	486	0%	100%	0%	0%	0%	100%
428	100%	0%	100%	0%	0%	0%	487	9%	91%	0%	0%	100%	0%
429	100%	0%	86%	0%	9%	5%	488	0%	100%	0%	0%	100%	0%
430	100%	0%	97%	0%	0%	0%	489	0%	100%	0%	0%	100%	0%
431	99%	1%	93%	0%	7%	0%	490	0%	100%	10%	0%	20%	70%
432	100%	0%	100%	0%	0%	0%	491	3%	97%	0%	0%	100%	0%
433	100%	0%	81%	0%	1%	17%	492	0%	100%	0%	0%	100%	0%

	*Structure*		BAPS					*Structure*		BAPS			
ST	Pop1	Pop2	Pop1b	Pop1a	Pop2a	Pop2b	ST	Pop1	Pop2	Pop1a	Pop1b	Pop2a	Pop2b
493	1%	99%	0%	0%	100%	0%	551	0%	100%	0%	0%	0%	100%
494	0%	100%	0%	0%	32%	68%	552	100%	0%	100%	0%	0%	0%
495	0%	100%	0%	0%	0%	100%	553	99%	1%	96%	0%	1%	1%
496	0%	100%	0%	0%	0%	100%	554	100%	0%	100%	0%	0%	0%
497	9%	91%	36%	0%	0%	64%	555	98%	2%	68%	0%	21%	11%
498	4%	96%	0%	0%	80%	20%	556	100%	0%	100%	0%	0%	0%
499	0%	100%	0%	0%	100%	0%	557	100%	0%	100%	0%	0%	0%
500	0%	100%	4%	0%	0%	96%	558	100%	0%	100%	0%	0%	0%
501	4%	96%	2%	0%	98%	0%	559	100%	0%	100%	0%	0%	0%
502	0%	100%	0%	0%	0%	100%	560	100%	0%	100%	0%	0%	0%
503	0%	100%	0%	0%	100%	0%	561	100%	0%	100%	0%	0%	0%
504	0%	100%	0%	0%	99%	1%	562	0%	100%	0%	0%	42%	58%
505	0%	100%	0%	0%	0%	100%	563	100%	0%	91%	0%	9%	0%
506	0%	100%	0%	0%	0%	100%	564	100%	0%	100%	0%	0%	0%
507	0%	100%	0%	0%	0%	100%	565	100%	0%	99%	0%	0%	0%
508	0%	100%	0%	0%	0%	100%	566	100%	0%	88%	0%	11%	0%
509	0%	100%	0%	0%	97%	3%	567	100%	0%	84%	0%	16%	0%
510	0%	100%	0%	0%	0%	100%	568	99%	1%	100%	0%	0%	0%
511	0%	100%	0%	0%	0%	100%	569	100%	0%	100%	0%	0%	0%
512	0%	100%	0%	0%	0%	100%	570	100%	0%	100%	0%	0%	0%
513	0%	100%	0%	0%	0%	100%	571	100%	0%	85%	0%	15%	0%
514	93%	7%	46%	0%	54%	0%	572	100%	0%	100%	0%	0%	0%
515	100%	0%	87%	0%	13%	0%	573	100%	0%	99%	0%	0%	0%
516	100%	0%	100%	0%	0%	0%	574	100%	0%	82%	0%	17%	1%
517	100%	0%	99%	0%	0%	1%	575	100%	0%	99%	0%	0%	0%
518	11%	89%	1%	0%	99%	0%	576	100%	0%	100%	0%	0%	0%
519	0%	100%	0%	0%	100%	0%	577	100%	0%	100%	0%	0%	0%
520	0%	100%	0%	0%	100%	0%	578	100%	0%	100%	0%	0%	0%
521	0%	100%	0%	0%	100%	0%	579	100%	0%	100%	0%	0%	0%
522	0%	100%	0%	0%	0%	100%	580	98%	2%	86%	0%	0%	14%
523	0%	100%	10%	0%	0%	89%	581	100%	0%	100%	0%	0%	0%
524	3%	97%	20%	0%	19%	61%	582	100%	0%	100%	0%	0%	0%
525	5%	95%	14%	0%	86%	0%	583	100%	0%	100%	0%	0%	0%
526	0%	100%	0%	0%	100%	0%	584	100%	0%	100%	0%	0%	0%
527	0%	100%	0%	0%	0%	100%	585	100%	0%	100%	0%	0%	0%
528	2%	98%	31%	0%	0%	69%	586	100%	0%	100%	0%	0%	0%
529	0%	100%	0%	0%	89%	11%	587	100%	0%	100%	0%	0%	0%
530	0%	100%	0%	0%	0%	100%	588	100%	0%	95%	0%	1%	4%
531	0%	100%	0%	0%	0%	100%	589	0%	100%	0%	0%	85%	15%
532	0%	100%	0%	0%	0%	100%	591	99%	1%	83%	0%	17%	0%
533	0%	100%	0%	0%	4%	96%	592	99%	2%	48%	0%	48%	4%
534	0%	100%	0%	0%	72%	28%	593	91%	9%	1%	0%	99%	0%
535	0%	100%	0%	0%	96%	4%	594	70%	30%	39%	0%	61%	0%
536	0%	100%	0%	0%	100%	0%	595	100%	0%	98%	0%	0%	0%
538	66%	34%	1%	0%	99%	0%	596	97%	4%	0%	0%	99%	0%
539	0%	100%	0%	0%	100%	0%	597	100%	0%	100%	0%	0%	0%
540	0%	100%	0%	0%	0%	100%	598	100%	0%	95%	0%	5%	0%
541	0%	100%	0%	0%	0%	100%	599	100%	0%	99%	0%	1%	0%
542	0%	100%	0%	0%	4%	96%	600	100%	0%	100%	0%	0%	0%
543	0%	100%	0%	0%	59%	41%	601	94%	6%	53%	0%	8%	39%
544	0%	100%	0%	0%	0%	100%	602	100%	0%	99%	0%	1%	0%
545	0%	100%	0%	0%	22%	78%	603	100%	0%	100%	0%	0%	0%
546	0%	100%	16%	0%	0%	84%	604	100%	0%	76%	0%	23%	1%
547	0%	100%	0%	0%	1%	99%	605	100%	0%	97%	0%	3%	0%
548	0%	100%	0%	0%	100%	0%	606	100%	0%	100%	0%	0%	0%
549	0%	100%	0%	0%	0%	100%	607	100%	0%	100%	0%	0%	0%
550	5%	95%	17%	0%	83%	0%	608	100%	0%	100%	0%	0%	0%

	*Structure*		BAPS					*Structure*		BAPS			
ST	Pop1	Pop2	Pop1b	Pop1a	Pop2a	Pop2b	ST	Pop1	Pop2	Pop1a	Pop1b	Pop2a	Pop2b
609	23%	77%	1%	0%	98%	1%	668	100%	0%	100%	0%	0%	0%
610	100%	0%	100%	0%	0%	0%	670	0%	100%	0%	0%	100%	0%
611	100%	0%	100%	0%	0%	0%	671	49%	51%	23%	0%	75%	1%
612	97%	3%	64%	0%	1%	35%	672	0%	100%	0%	0%	57%	43%
613	100%	0%	79%	0%	0%	20%	673	100%	0%	89%	0%	11%	0%
614	100%	0%	84%	0%	0%	16%	674	100%	0%	100%	0%	0%	0%
616	92%	8%	81%	0%	0%	19%	675	100%	0%	100%	0%	0%	0%
617	100%	0%	100%	0%	0%	0%	676	99%	1%	100%	0%	0%	0%
618	98%	2%	54%	0%	40%	6%	677	14%	86%	0%	0%	46%	54%
619	100%	0%	52%	0%	48%	0%	678	100%	0%	96%	0%	0%	4%
620	100%	0%	100%	0%	0%	0%	679	100%	0%	100%	0%	0%	0%
621	100%	0%	63%	0%	37%	0%	680	100%	0%	65%	0%	35%	0%
622	87%	13%	2%	0%	98%	0%	681	100%	0%	100%	0%	0%	0%
623	100%	0%	70%	0%	2%	28%	682	87%	13%	69%	0%	0%	31%
624	100%	0%	100%	0%	0%	0%	683	100%	0%	99%	0%	0%	0%
625	100%	0%	100%	0%	0%	0%	684	0%	100%	0%	0%	0%	100%
626	100%	0%	100%	0%	0%	0%	685	0%	100%	0%	0%	55%	45%
627	100%	0%	100%	0%	0%	0%	686	0%	100%	12%	0%	0%	88%
628	100%	0%	99%	0%	0%	0%	687	0%	100%	0%	0%	0%	100%
629	100%	0%	100%	0%	0%	0%	688	1%	99%	0%	0%	99%	1%
630	100%	0%	100%	0%	0%	0%	689	1%	99%	0%	0%	100%	0%
631	100%	0%	99%	0%	0%	0%	690	0%	100%	0%	0%	75%	25%
632	100%	0%	96%	0%	4%	0%	691	1%	100%	19%	0%	21%	59%
633	100%	0%	96%	0%	0%	0%	692	28%	72%	10%	0%	90%	0%
634	100%	0%	99%	0%	0%	0%	693	0%	100%	0%	0%	0%	100%
635	100%	0%	98%	0%	2%	0%	694	0%	100%	0%	0%	23%	77%
636	100%	0%	41%	59%	0%	0%	695	11%	89%	44%	0%	0%	55%
637	100%	0%	100%	0%	0%	0%	697	100%	0%	66%	0%	2%	32%
638	100%	0%	94%	0%	2%	4%	698	2%	98%	0%	79%	21%	0%
639	100%	0%	100%	0%	0%	0%	700	79%	21%	32%	0%	68%	0%
640	100%	0%	100%	0%	0%	0%	701	0%	100%	0%	0%	10%	90%
641	100%	0%	100%	0%	0%	0%	702	1%	99%	0%	0%	100%	0%
642	99%	1%	74%	0%	2%	24%	703	2%	98%	3%	0%	97%	0%
643	100%	0%	100%	0%	0%	0%	704	1%	99%	0%	0%	64%	36%
644	100%	0%	100%	0%	0%	0%	705	0%	100%	0%	0%	0%	100%
645	100%	0%	94%	0%	0%	6%	707	3%	97%	0%	0%	99%	1%
647	90%	10%	6%	0%	81%	13%	708	0%	100%	2%	0%	20%	78%
648	100%	0%	94%	0%	4%	2%							
649	0%	100%	0%	0%	100%	0%							
650	0%	100%	0%	0%	20%	80%							
651	1%	99%	0%	0%	81%	19%							
652	0%	100%	0%	0%	71%	29%							
653	0%	100%	0%	0%	100%	0%							
654	0%	100%	0%	0%	0%	100%							
655	1%	99%	0%	0%	100%	0%							
656	1%	99%	0%	0%	100%	0%							
657	0%	100%	0%	0%	100%	0%							
658	0%	100%	0%	0%	0%	100%							
659	5%	95%	0%	0%	100%	0%							
660	100%	0%	75%	0%	12%	12%							
661	0%	100%	0%	0%	98%	2%							
662	5%	95%	0%	0%	99%	0%							
663	100%	0%	57%	0%	0%	43%							
664	100%	0%	94%	0%	6%	0%							
665	100%	0%	100%	0%	0%	0%							
666	100%	0%	96%	0%	1%	2%							
667	100%	0%	100%	0%	0%	0%							

While we present here a list of STs and the likelihood of assignment into each population, we recognize that new STs will be found with future sampling, limiting the long-term utility of our analyses. However, due to the relatively low diversity and high recombination rates relative to mutation in *B. pseudomallei* [11], it is likely that many new STs will not contain novel alleles, but rather will comprise new combinations of characterized alleles. As population assignments with *Structure* are based on allele frequencies in a population, we include this information here with the expectation that this resource will continue to be useful even as novel STs are discovered (Figure S3 and Table S1). We suggest that alleles that are predominantly associated with population 1 or population 2 can be used to estimate population assignment for novel STs. Of 50 randomly selected STs, all but three could be assigned based on the presence of alleles predominantly associated with one population (≥95% of their occurrence is attributed to one population). These three STs do not have a high affinity to either populations as all were originally assigned with <95% confidence by *Structure* and BAPS. Of the 664 *B. pseudomallei* STs, 80% have alleles that are exclusively found in one of the two main populations and 93% have alleles that are associated with one of these populations in ≥95% of their occurrences. Thus for new STs, allele frequency data can shed light on appropriate population assignments.

As lateral gene transfer is increasingly found to play an important role in the population dynamics of a range of bacterial species, population genetics tools such as *Structure* and BAPS will become more widely used by epidemiologists. The approach described here facilitates rapid assignment of isolates to established populations without needing to compile data, or learn and run a new application. Population assignment is one of the first steps in epidemiological tracking of disease and can be used to identify and track bacterial introductions into new regions. We have expanded on our previous work [11] by rigorously exploring the composition of the two major populations of *B. pseudomallei*. Our results suggest that the programs *Structure* and BAPS are both sensitive and accurate for population assignment of *B. pseudomallei* using MLST data, as the two programs provide similar results. The relative rate of recombination to mutation at MLST loci for *B. pseudomallei* is higher than for any other bacterial species yet reported [11], meaning that allele frequency differences among populations is an appropriate method for determining population structure. Examining allele frequencies when deciphering population structure is standard for eukaryotes, where high recombination rates cause allelic frequency differences among populations through genetic drift [29].

Population assignment is an important aspect of epidemiological and forensic attribution. As knowledge of population dynamics and geographical distribution of a species increases, attribution can be attempted at an increasingly fine scale, allowing investigators to focus their attention on a very small and well-defined population and geographic region. For *B. pseudomallei*, little is currently known about population dynamics, evolution and even geographical distribution. High relative rates of recombination to mutation complicate attempts to discern population structure for this species using strictly phylogenetic approaches. MLST analyses are popular for bacterial pathogens and the large data set collected for *B. pseudomallei* has allowed for the robust identification of two main populations that correspond to the endemic geographical regions of Southeast Asia and Australia. While substructure within these two populations likely exists, such as the third population identified by BAPS, the seven MLST genes and the current set of STs do not provide enough resolution for further robust differentiation among subpopulations. Genotype interrogation at more loci or great numbers of STs will increase our knowledge of subpopulation dynamics, but in the meantime our current ability to differentiate between the two or three major populations is an important first step for epidemiological attribution. Increasing knowledge of the geographic distribution and population structure of *B. pseudomallei* STs form the foundation for future work on the evolution, population dynamics and geographical distribution of subpopulations of this bacterium.

## Supporting Information

Figure S1 **Comparison of K values from** ***Structure*** **using both ‘admixture’ and ‘no admixture’ models.** (A) Log likelihood and average within run variance associated with different values of *K*, (B) Log likelihood of different values of *K*, (C) *ΔK* for different values of *K*. Importantly, in this figure it should be noted that the most likely value is *K* = 2.
(PDF)

Figure S2 **Comparison of** ***K*** **values from** ***Structure*** **runs using the ‘no admixture’ model.** (A) Log likelihood and average variance associated with different values of *K* from *Structure* along with results from calculating *ΔK* from these values and (B) plot showing the change in *ΔK* for each population of *K*. Analyses using both models show that *K* = 2 (i.e. two populations) is the most supported *K* value in the *B. pseudomallei* MLST dataset using *Structure*.
(PDF)

Figure S3 **Allele frequencies across 664 STs in each** ***B. pseudomallei*** **population.** The frequencies of alleles from STs assigned to each population based on *Structure* and BAPS are shown as a stacked bar graph. For BAPS data, Population 1a and Population 1b were combined as Population 1b only consisted of three STs. The red bar represents alleles placed in Population 1 (predominantly Australian STs) with ≥95% probability of assignment, the green bar represents alleles placed in Population 2 (*Structure*) and 2a (BAPS) (predominantly Southeast Asian STs) with ≥95% probability of assignment, the yellow bar represents alleles placed in Population 2b with probability of assignment by BAPS, and the error bars represents the number of alleles placed in a population with a probability of assignment <95%.
(PDF)

Table S1 **Allele frequencies across 664 STs in each** ***B. pseudomallei*** **population.**
(XLSX)

Text S1 **Calculating Δ** ***K*** **(adapted from Evanno et al.** [**17**] **).**
(DOC)
