# Supplementary figures and images for: Epidemiological Tracking and Population Assignment of the Non-Clonal Bacterium, Burkholderia pseudomallei
Source: PLoS Negl Trop Dis. 2011 Dec 13;5(12):e1381. doi: 10.1371/journal.pntd.0001381 (PMC3236730; doi:10.1371/journal.pntd.0001381)

A

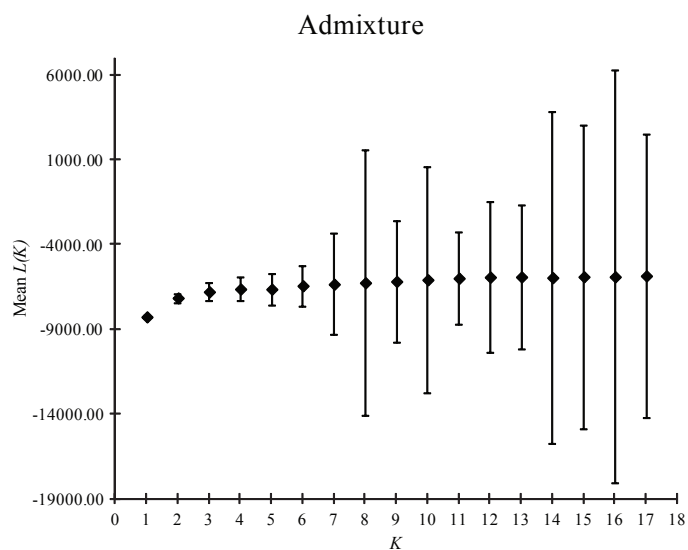

NO Admixture

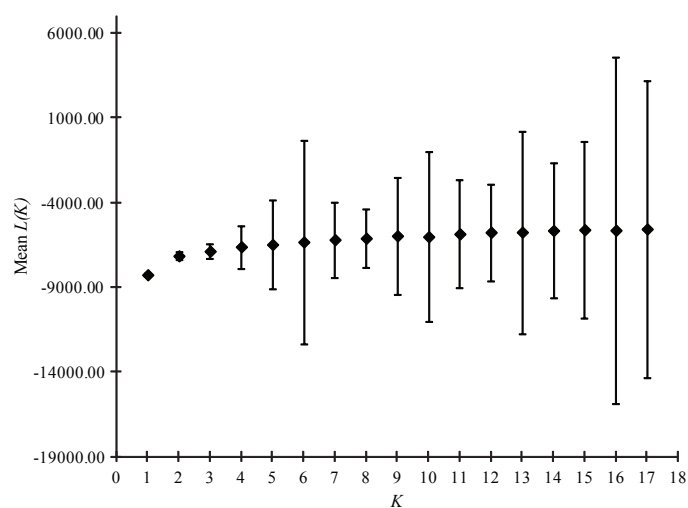

B

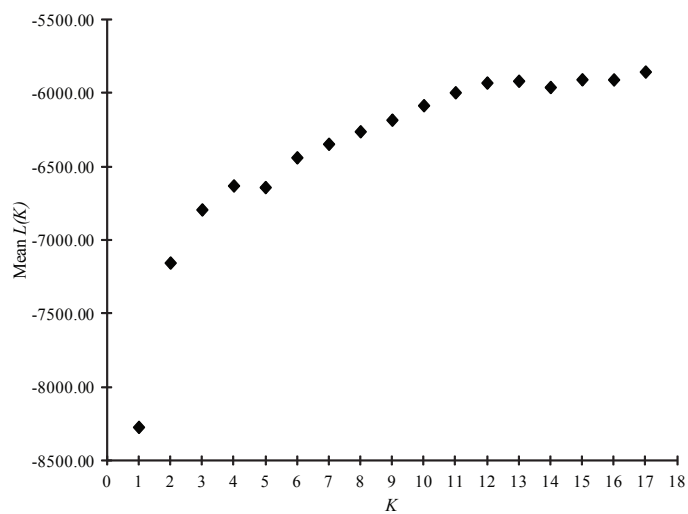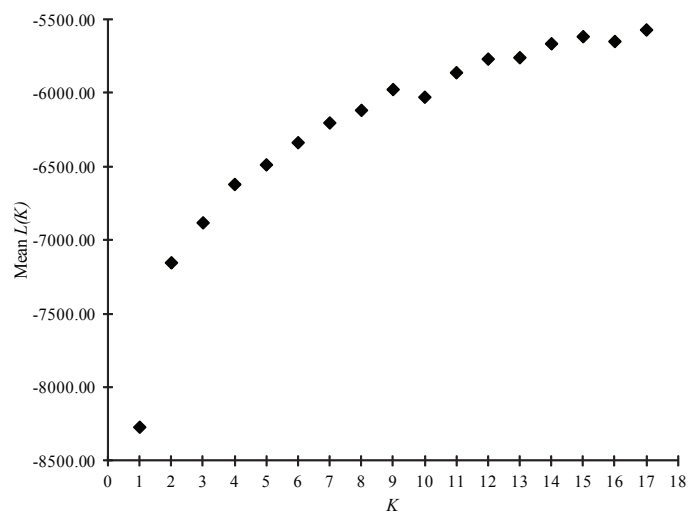

C

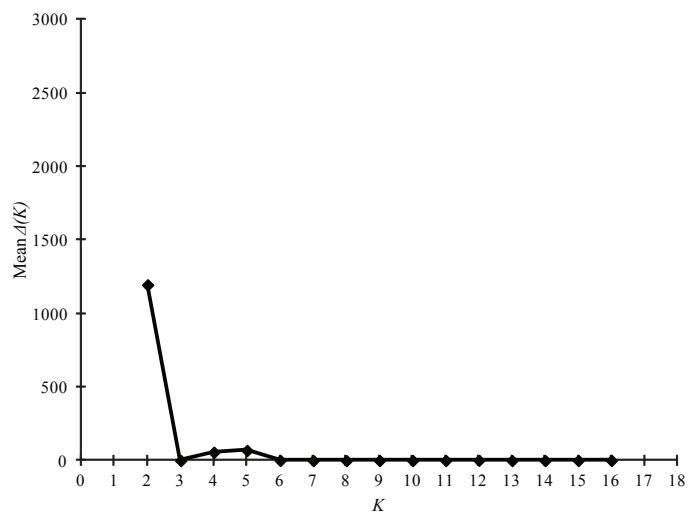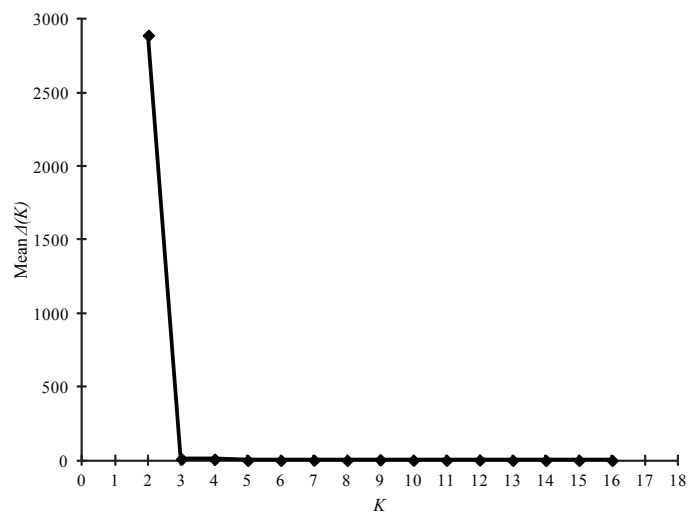

Supplement: Figure S1 — Comparison of K values from Structure using both ‘admixture’ and ‘no admixture’ models. (A) Log likelihood and average within run variance associated with different values of K, (B) Log likelihood of different values of K, (C) ΔK for different values of K. Importantly, in this figure it should be noted that the most likely value is K = 2. (PDF) [file pntd.0001381.s001.pdf]

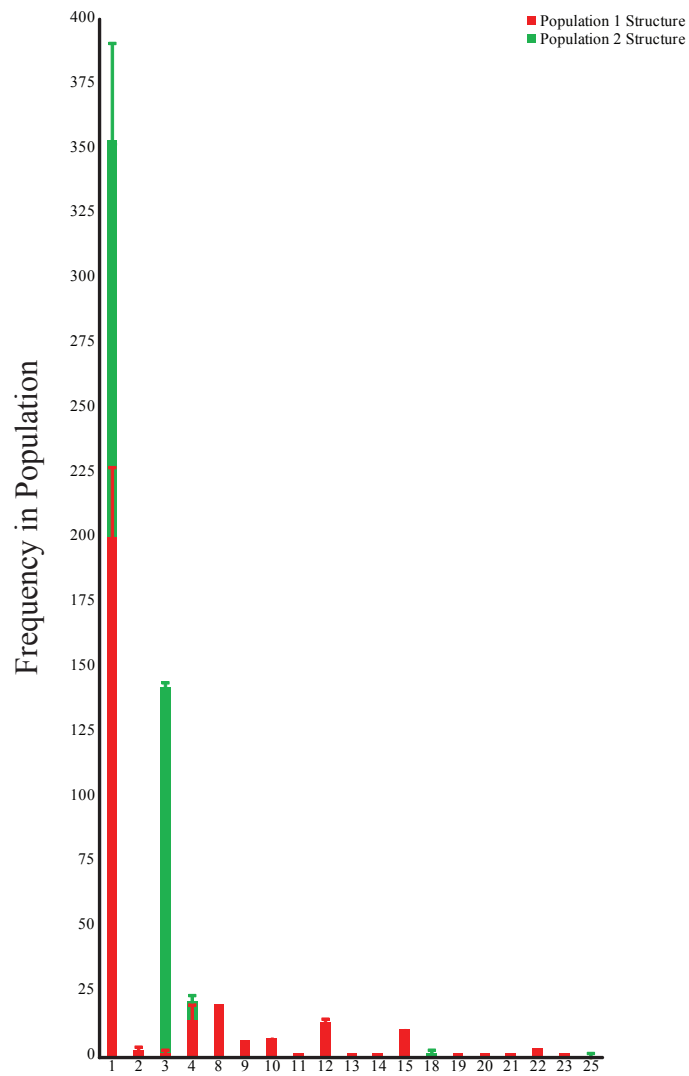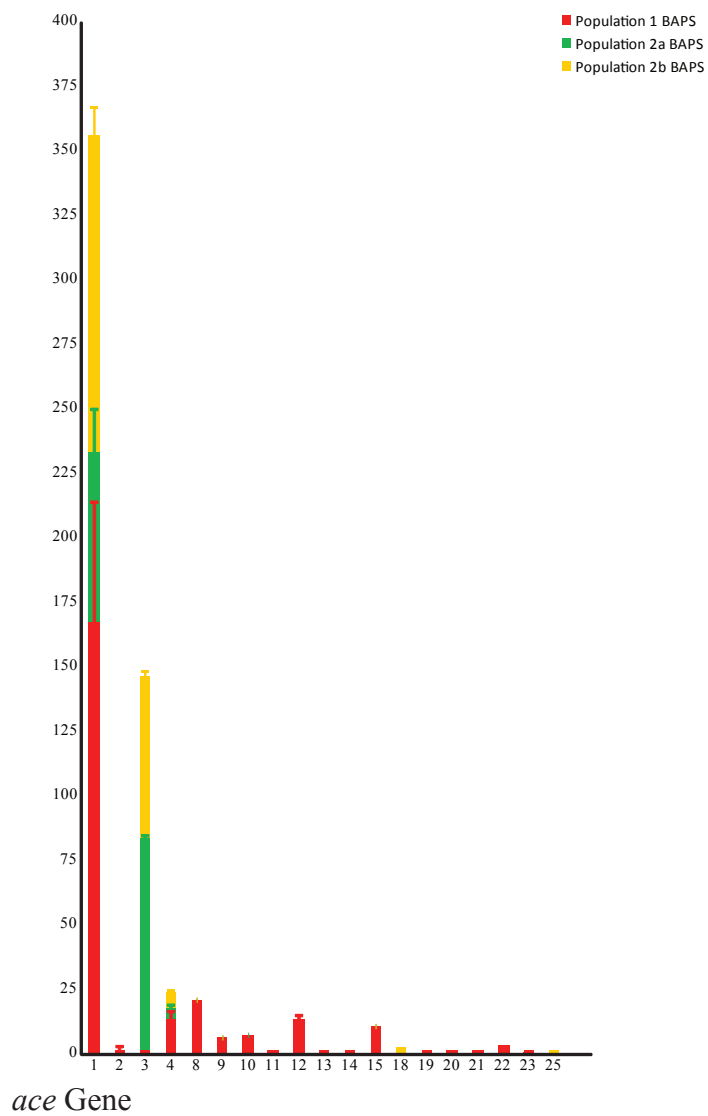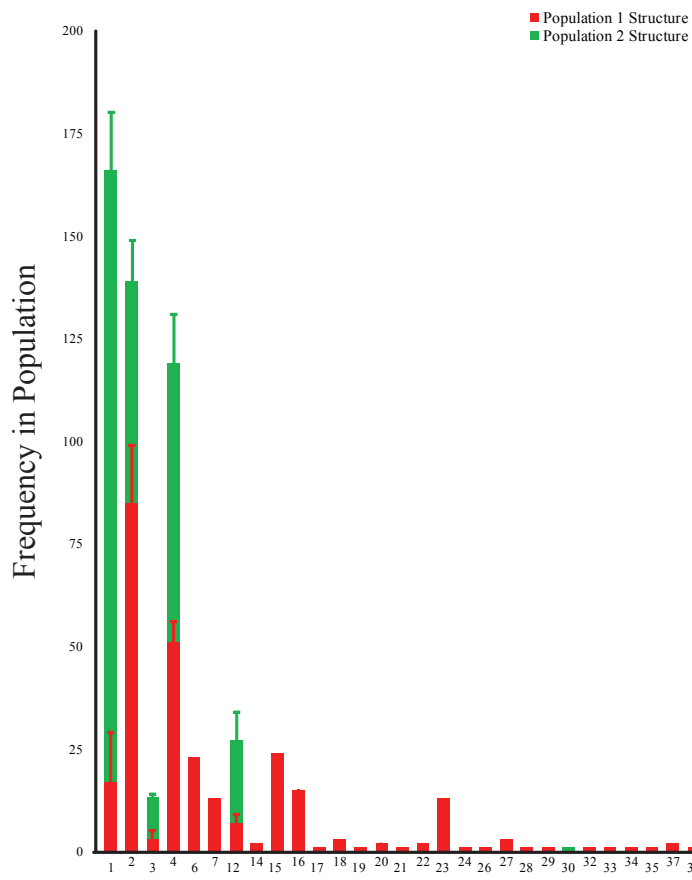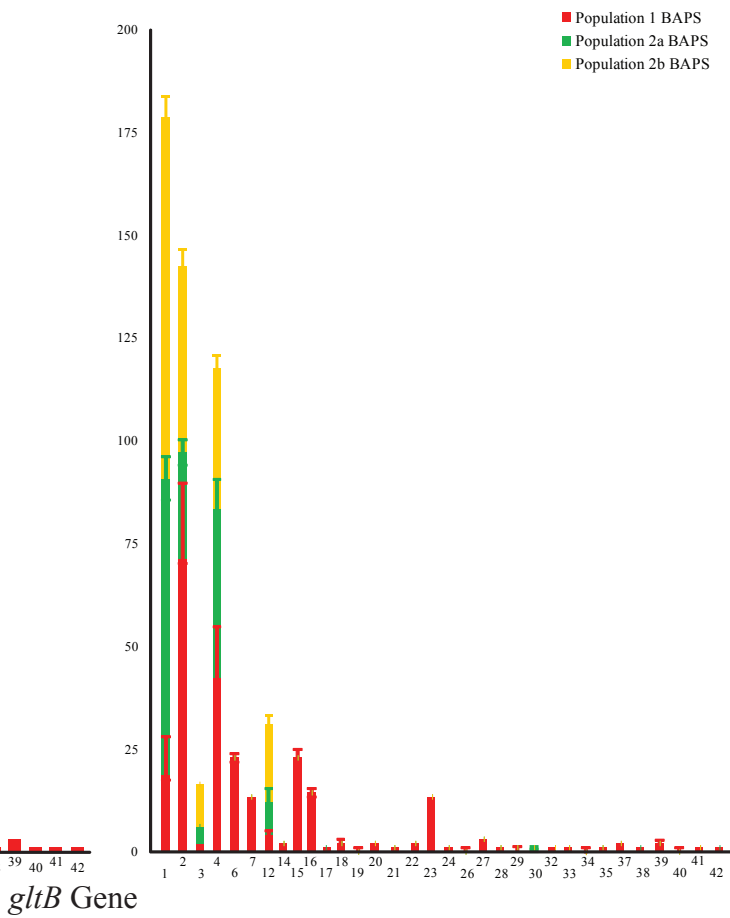

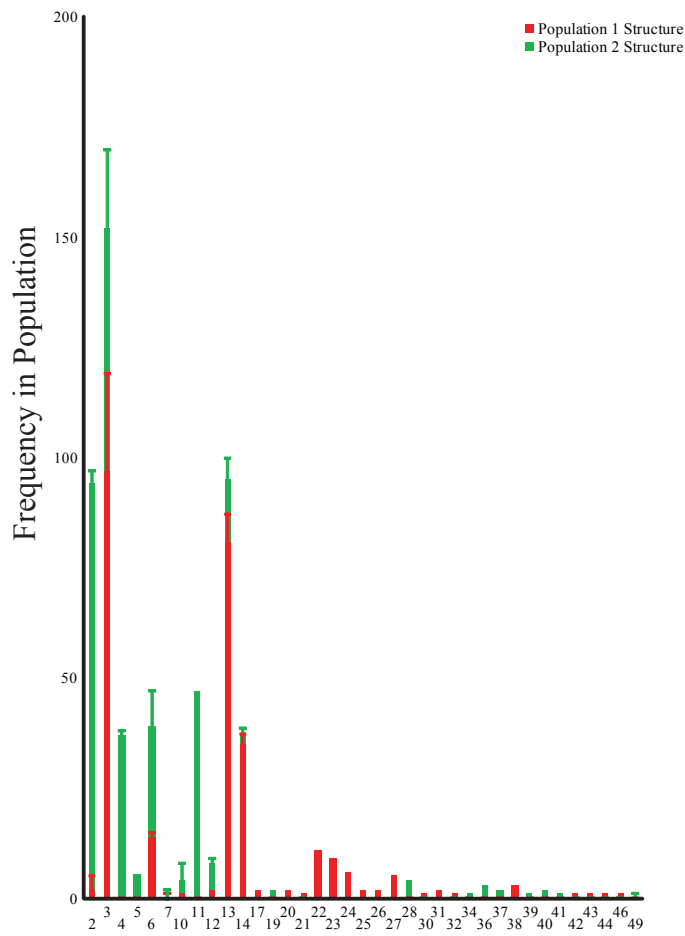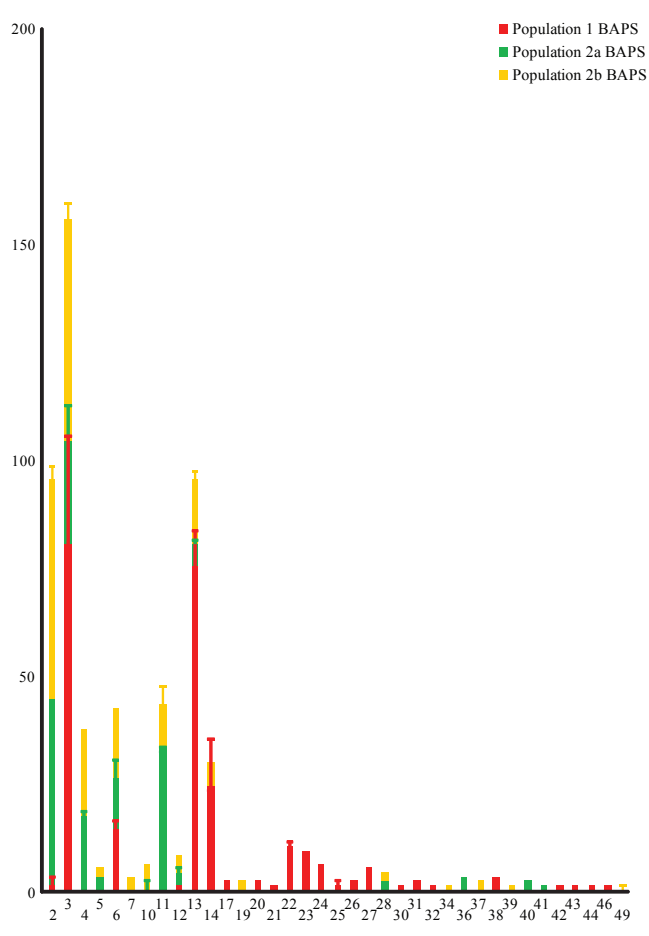

*gmhD* Gene

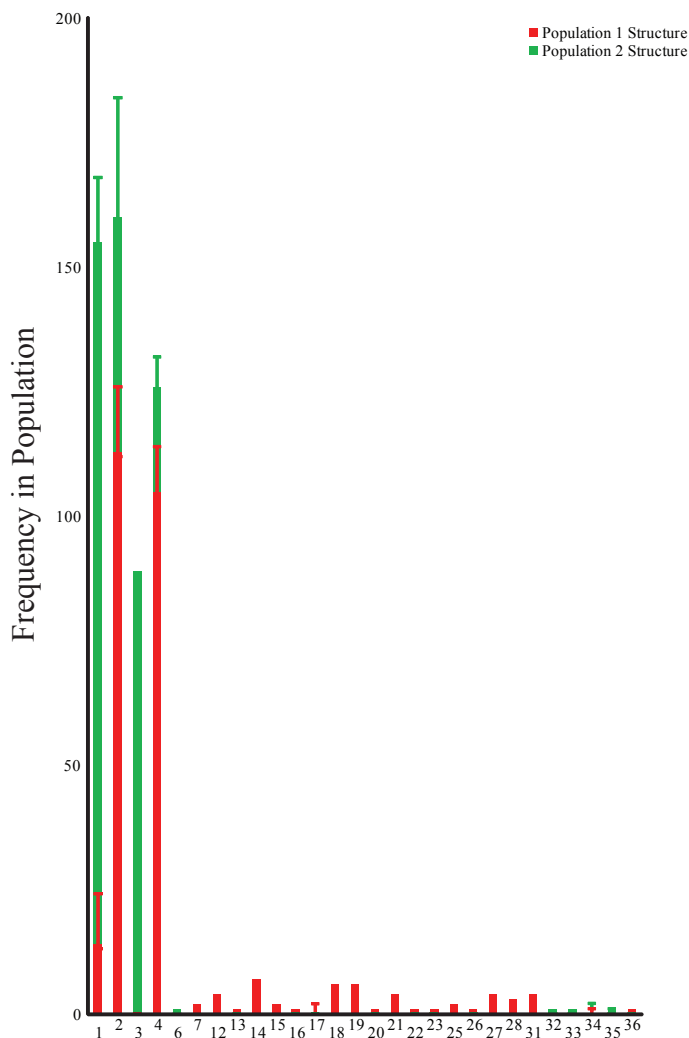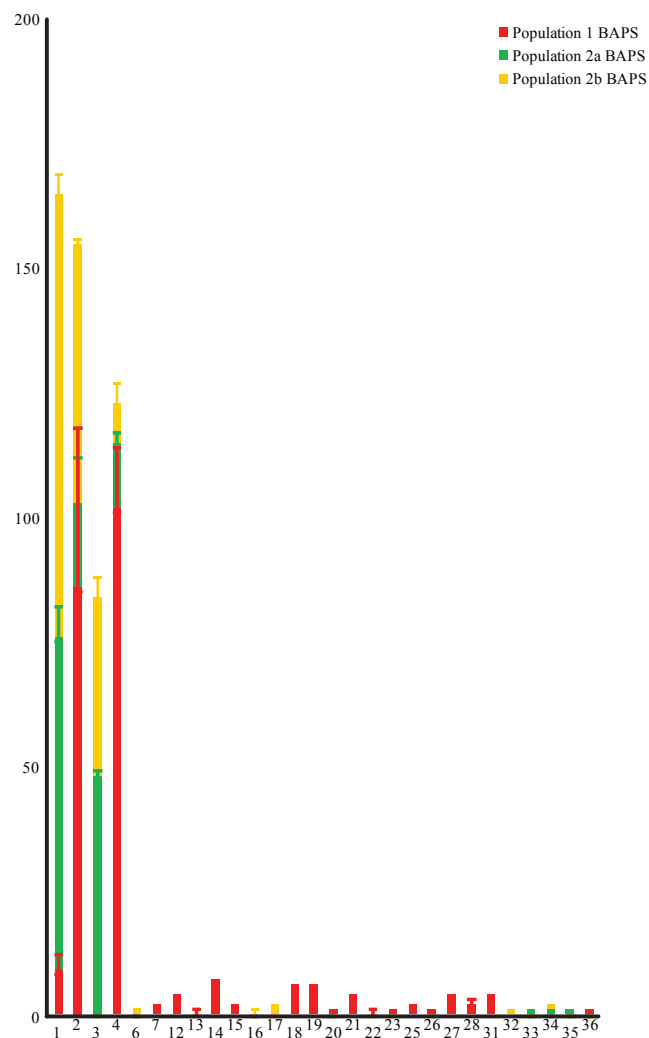

*lepA* Gene

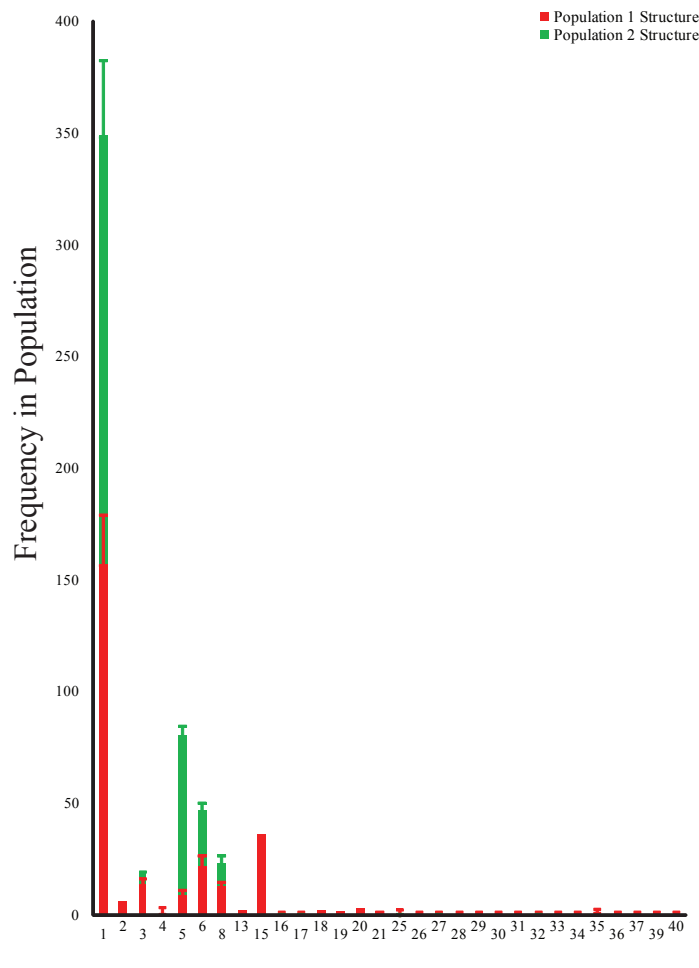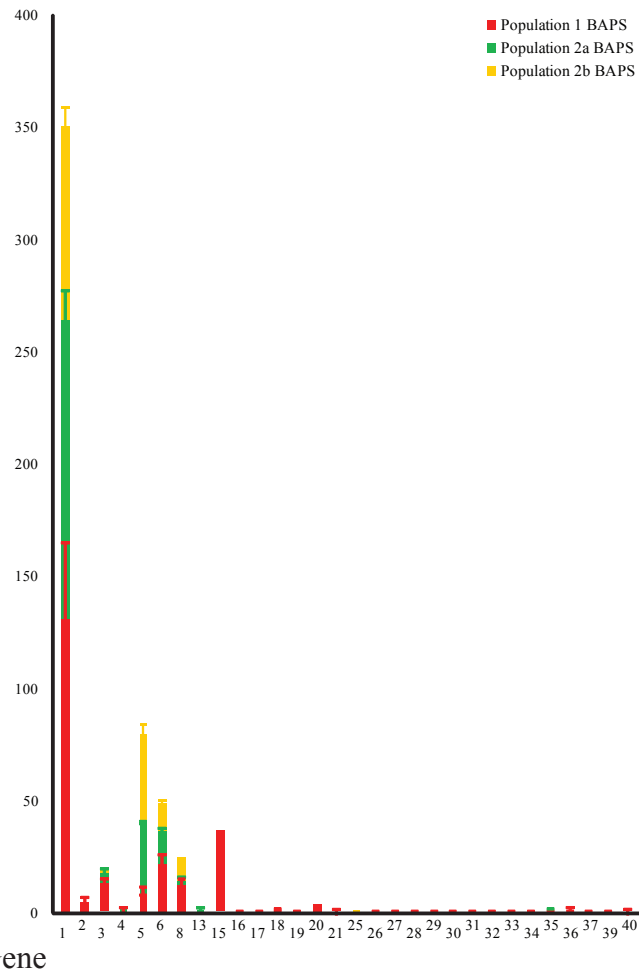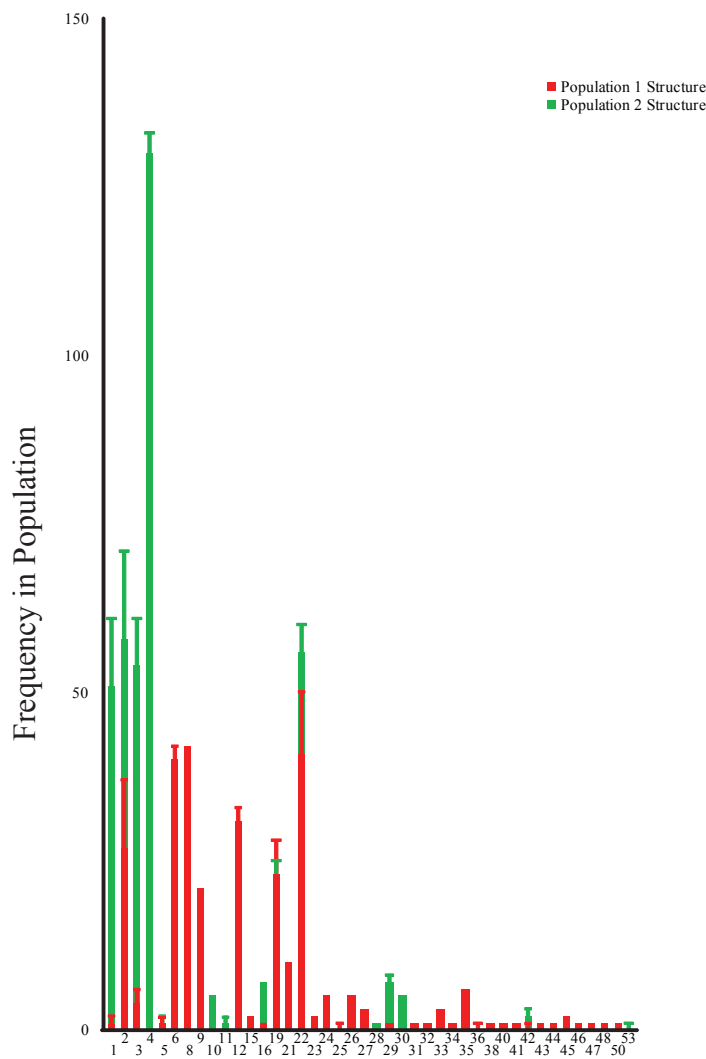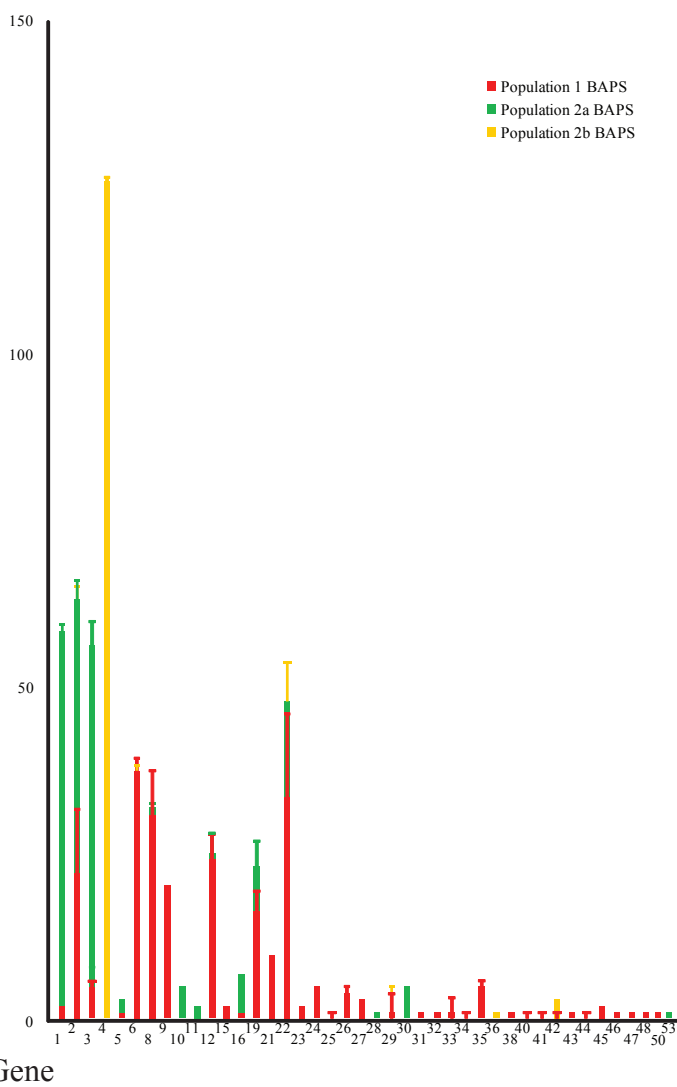

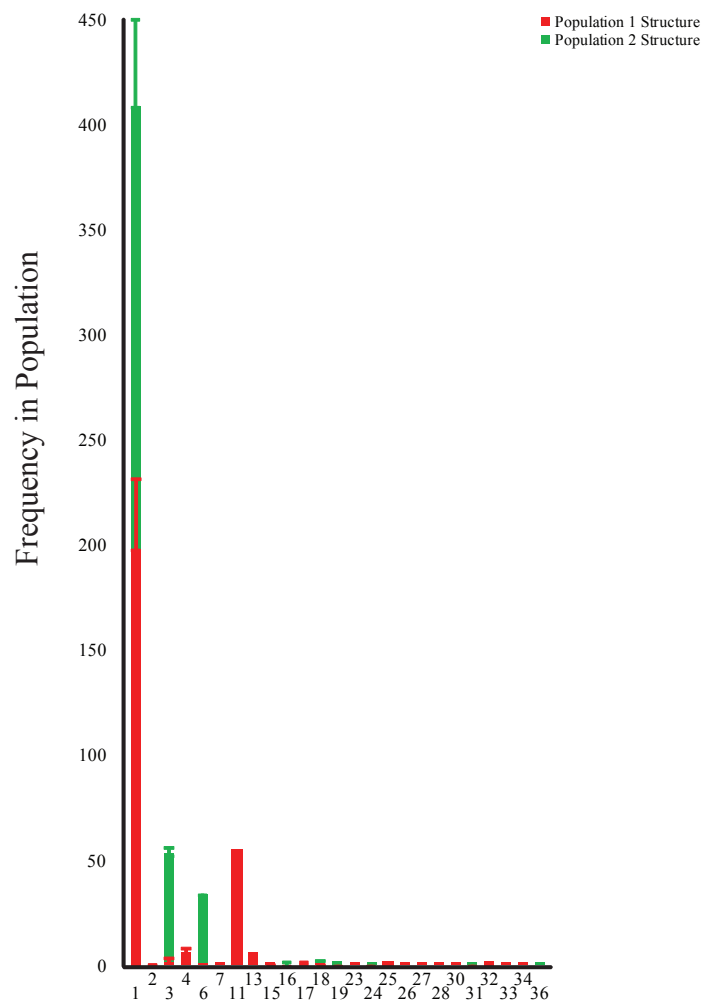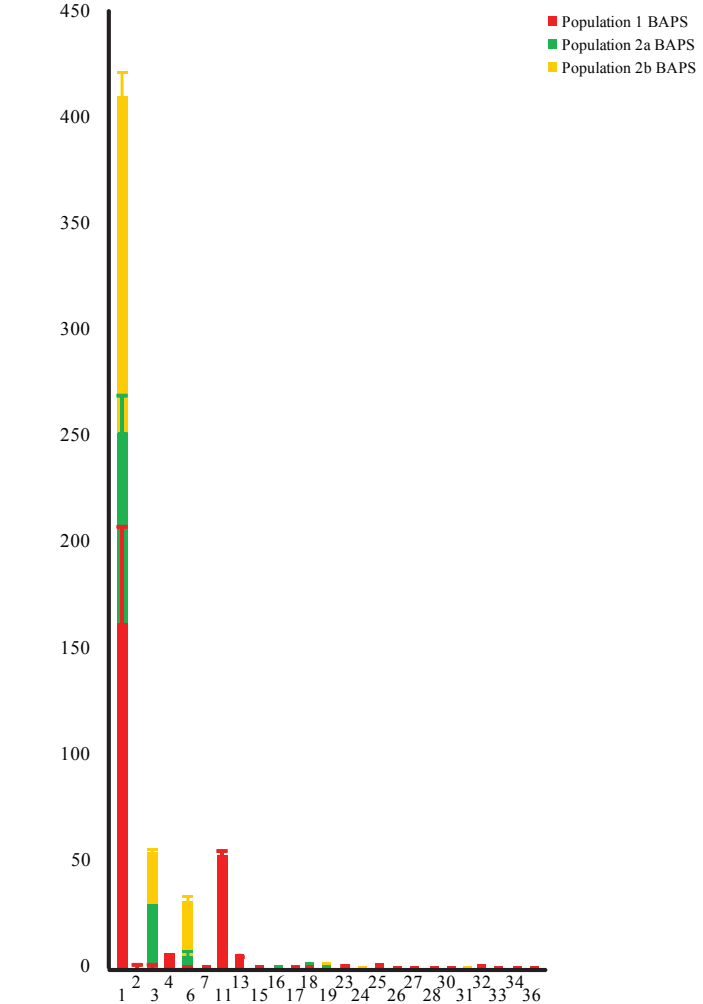

*ndh* Gene

Supplement: Figure S3 — Allele frequencies across 664 STs in each B. pseudomallei population. The frequencies of alleles from STs assigned to each population based on Structure and BAPS are shown as a stacked bar graph. For BAPS data, Population 1a and Population 1b were combined as Population 1b only consisted of three STs. The red bar represents alleles placed in Population 1 (predominantly Australian STs) with ≥95% probability of assignment, the green bar represents alleles placed in Population 2 (Structure) and 2a (BAPS) (predominantly Southeast Asian STs) with ≥95% probability of assignment, the yellow bar represents alleles placed in Population 2b with probability of assignment by BAPS, and the error bars represents the number of alleles placed in a population with a probability of assignment <95%. (PDF) [file pntd.0001381.s003.pdf]
