# Supplementary material for: Epidemiological Tracking and Population Assignment of the Non-Clonal Bacterium, Burkholderia pseudomallei
Source: PLoS Negl Trop Dis. 2011 Dec 13;5(12):e1381. doi: 10.1371/journal.pntd.0001381 (PMC3236730; doi:10.1371/journal.pntd.0001381)

A

K	Ln P(D) or *L*(*K*)	Variance of *L*(*K*) given by *Structure*	Standard Deviation of *L*(*K*)	First order rate of change (*L*′(K))	Second order rate of change (*L*″(K))	*ΔK*
1	-8272.8	98.60	0	n/a	n/a	n/a
1	-8272.8	99.00				
1	-8272.8	98.50				
Mean	-8272.80					
2	-7155.4	265.30	0.351188458	1117.43	521.1333333	1483.914
2	-7155.7	270.60				
2	-7155	265.10				
Mean	-7155.37					
3	-6889.20	450.60	579.7933885	596.30	668	1.152135
3	-6898.40	584.80				
3	-5889.60	442.30				
Mean	-6559.07					
4	-6635.5	751.70	4.110150039	-71.70	60.76666667	14.78454
4	-6628.1	684.60				
4	-6628.7	705.90				
Mean	-6630.77					
5	-6641.40	990.20	2.762245467	-10.93	213.2	77.18358
5	-6639.10	932.30				
5	-6644.6	849.30				
Mean	-6641.70					
6	-6484.7	1037.20	76.42096135	202.27	n/a	n/a
6	-6351.20	1559.10				
6	-6482.40	972.80				
Mean	-6439.43					

B

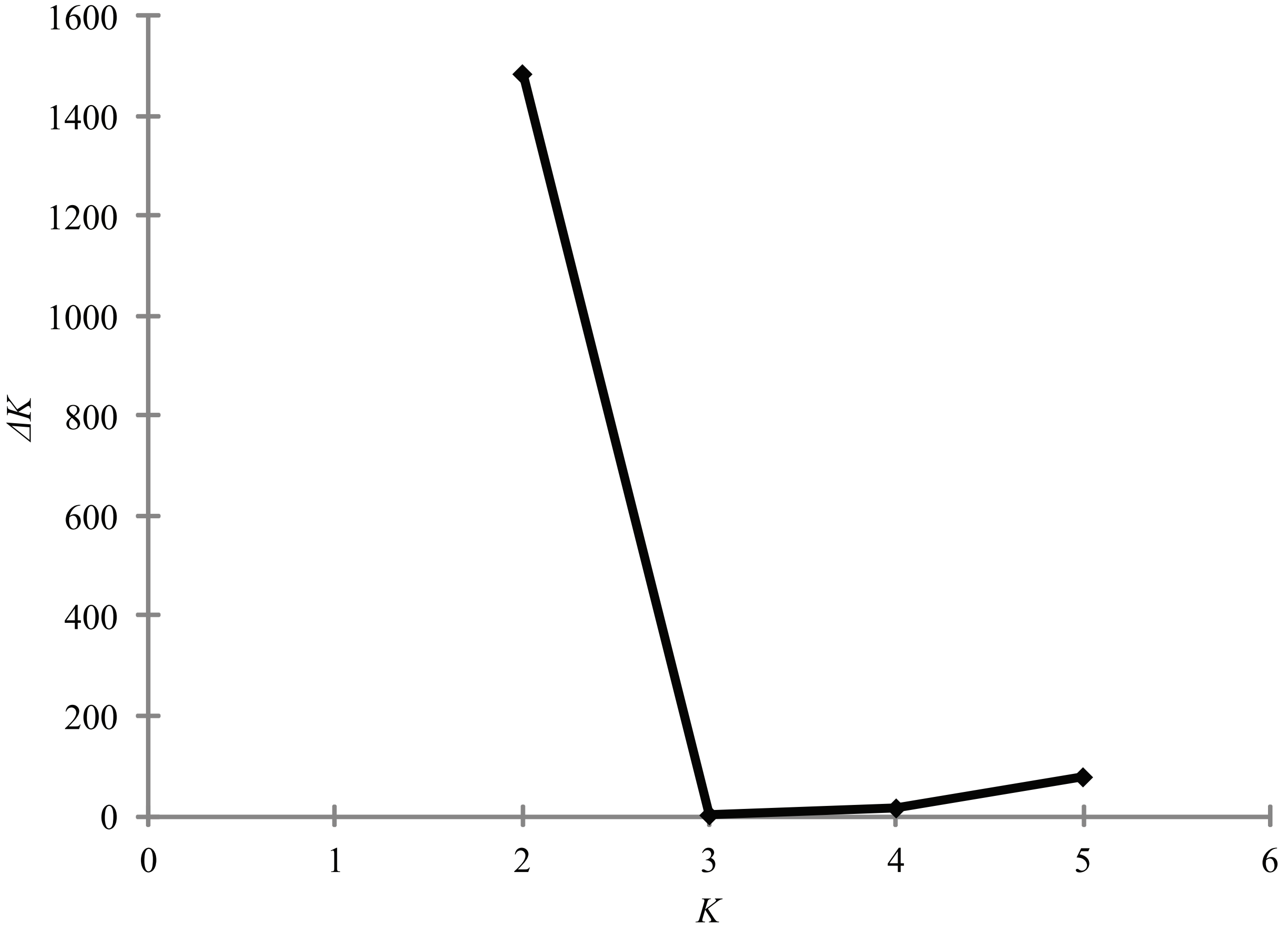

Supplement: Figure S2 — Comparison of K values from Structure runs using the ‘no admixture’ model. (A) Log likelihood and average variance associated with different values of K from Structure along with results from calculating ΔK from these values and (B) plot showing the change in ΔK for each population of K. Analyses using both models show that K = 2 (i.e. two populations) is the most supported K value in the B. pseudomallei MLST dataset using Structure. (PDF) [file pntd.0001381.s002.pdf]
