# Supplementary material for: Epidemiological Tracking and Population Assignment of the Non-Clonal Bacterium, Burkholderia pseudomallei
Source: PLoS Negl Trop Dis. 2011 Dec 13;5(12):e1381. doi: 10.1371/journal.pntd.0001381 (PMC3236730; doi:10.1371/journal.pntd.0001381)
Supplement: Text S1 — Calculating Δ K (adapted from Evanno et al. [17] ). (DOC) [file pntd.0001381.s005.doc]

**Text S1: Calculating ∆*K* (adapted from Evanno et al. [17])**

1) Calculate the mean of all Ln P(D) from *Structure* for all runs at each population of K. Using Evanno et al. [17] nomenclature, Ln P(D) is referred to as *L*(*K*):

*m*(Ln P(D)) = *m*(*L*(*K*))

2) Calculate the standard deviation of *L*(*K*) at each *K* population(s):

*s*(*L*(*K*))

3) Calculate the first order rate of change (*L'*(*K*)) as the mean *L*(*K*) value at *K* population(s) minus the mean *L*(*K*) value at *K* − 1 population(s):

*L'*(*K*) = *L*(*K*) − *L*(*K* − 1)

4) Calculate the second order rate of change (*L"*(*K*)) as the absolute value of the result of subtracting the first order rate of change at population *K* from the first order rate of change at *K* + 1 populations:

|*L"*(*K*)| = |*L'*(*K* + 1) − *L'*(*K*)|

5) Calculate *ΔK* as the absolute value of the second order reaction rate of change for *K* population(s), divided by the standard deviation of *L*(*K*) (calculated in step 2 above; see figure S2 for an example using 3 runs at K = 2 through 6):

*ΔK* = |*L"*(*K*)| ÷ *s*[*L*(*K*)]
